# GLUT3 induced by AMPK/CREB1 axis is key for withstanding energy stress and augments the efficacy of current colorectal cancer therapies

**DOI:** 10.1038/s41392-020-00220-9

**Published:** 2020-09-02

**Authors:** Weixing Dai, Ye Xu, Shaobo Mo, Qingguo Li, Jun Yu, Renjie Wang, Yanlei Ma, Yan Ni, Wenqiang Xiang, Lingyu Han, Long Zhang, Sanjun Cai, Jun Qin, Wen-Lian Chen, Wei Jia, Guoxiang Cai

**Affiliations:** 1 Department of Colorectal Surgery, Fudan University Shanghai Cancer Center, Shanghai, China; 2 Department of Oncology, Shanghai Medical College, Fudan University, Shanghai, China; 3 Department of Surgery, Johns Hopkins University School of Medicine, Baltimore, MD 21287 USA; 4 The Children’s Hospital, School of Medicine, Zhejiang University, Zhejiang, China; 5 The Key Laboratory of Stem Cell Biology, CAS Center for Excellence in Molecular Cell Science, Institute of Health Sciences, Shanghai Institutes for Biological Sciences, Chinese Academy of Sciences, Shanghai, China; 6 Cancer Institute, Longhua Hospital, Shanghai University of Traditional Chinese Medicine, Shanghai, 200032 China; 7 University of Hawaii Cancer Center, Honolulu, HI 96813 USA; 8 School of Chinese Medicine, Hong Kong Baptist University, Kowloon Tong, Hong Kong China

**Keywords:** Cancer metabolism, Epigenetics

## Abstract

Cancer cells are usually characterized by hyperactive glucose metabolism, which can often lead to glucose scarcity; thus, alternative pathways to rewire cancer metabolism are required. Here, we demonstrated that GLUT3 was highly expressed in colorectal cancer (CRC) and negatively linked to CRC patient outcomes, whereas GLUT1 was not associated with CRC prognosis. Under glucose-limiting conditions, GLUT3 expedited CRC cell growth by accelerating glucose input and fuelling nucleotide synthesis. Notably, GLUT3 had a greater impact on cell growth than GLUT1 under glucose-limiting stress. Mechanistically, low-glucose stress dramatically upregulated GLUT3 via the AMPK/CREB1 pathway. Furthermore, high GLUT3 expression remarkably increased the sensitivity of CRC cells to treatment with vitamin C and vitamin C-containing regimens. Together, the results of this study highlight the importance of the AMPK/CREB1/GLUT3 pathway for CRC cells to withstand glucose-limiting stress and underscore the therapeutic potential of vitamin C in CRC with high GLUT3 expression.

## Introduction

Colorectal cancer (CRC) is the third most prevalent cancer and the fourth most common cause of cancer death worldwide.^1,2^ The past decades have witnessed a significant improvement in the prognosis of patients with CRC.^3^ However, ~25% of these patients are diagnosed with CRC at an advanced stage or with metastasis; the 5-year survival of this subgroup is <20%, and only limited targeted therapies are available.^4^ Thus, there is a continued need to comprehensively understand the underlying mechanisms that promote CRC progression to identify new therapeutic targets.

Cancer cells share a common phenotype that includes efficient energy generation and the synthesis of macromolecules required for their neoplastic growth.^5,6^ To meet this anabolic demand, cancer cells usually adopt augmented nutrient acquisition strategies coupled with increased flux through downstream anabolic pathways. This unique metabolic dependence distinguishes cancer cells from their normal cellular counterparts. Indeed, metabolic reprogramming during tumorigenesis is an essential process in nearly all cancer cells.^7^ Of note, the Warburg effect, discovered by Otto Warburg in the 1920s, is the first example of metabolic reprogramming, as it describes the notion that cancer cells tend to undergo increased glycolysis rather than mitochondrial oxidative phosphorylation (OXPHOS) to generate adenosine triphosphate (ATP), regardless of the availability of oxygen.^8^ This metabolic process, termed aerobic glycolysis, is now considered one of the 10 hallmarks of cancers.^9^

Tumour cells are characterized by ectopic glucose uptake because they require large amounts of glucose as a metabolic fuel for enhanced glycolytic flux, which provides not only energy but also crucial biomolecules essential for their survival, proliferation and invasion.^10^ The following four glucose transporters have been well studied: GLUT1, GLUT2, GLUT3 and GLUT4, which are encoded by *SLC2A1*, *SLC2A2*, *SLC2A3* and *SLC2A4*, respectively.^11,12^ GLUT1, also named glucose transporter member 1 (*SLC2A1*), has been demonstrated to be a predominant glucose transporter in nearly all cancer cells.^10^ Therefore, the elevated glucose uptake in cancers is thought to be primarily driven by GLUT1. Previous studies have confirmed *SLC2A1* as a prominent oncogenic gene that promotes remarkable enhancement of cell proliferation.^13,14^ GLUT2 is the principal transporter for transfer of glucose between liver and blood.^15^ Previous function analysis revealed that GLUT2 is mainly responsible for blood glucose monitoring and the control of pancreatic hormone secretion.^16^ A prognostic study conducted in liver cancer found that high expression of GLUT2 is associated with inferior survival of patients.^17^ However, the investigation of GLUT2 in CRC is scarce. GLUT4 is an insulin-regulated glucose transporter as well as an established downstream target of PI3K/AKT signalling axis contributing to the progression of cancers including CRC.^18^ Among the four glucose transporters, GLUT3 has the highest affinity for glucose.^19^ In contrast to the ubiquitous transporter GLUT1, physiological GLUT3 expression is largely restricted to cells that both exhibit a high glucose demand and reside in a glucose-poor microenvironment, such as brain tissue.^20,21^ Limited previous studies have focused on GLUT3, and scarce information about its prognostic role and oncogenic effect in CRC has been reported.^22,23^

Metabolic stress caused by limited energy supply during the process of rapid growth is common in solid tumours, including CRC.^24,25^ Glucose deficiency is one of the main patterns of metabolic stress because of the striking dependence of cancers on glucose as a carbon resource.^26^ Our preliminary bioinformatics analysis using public datasets demonstrated that the GLUT3-encoding gene *SLC2A3* was remarkably upregulated in CRC tissues and that high expression of *SLC2A3* but not *SLC2A1* was negatively associated with the overall survival of patients with CRC. Therefore, we hypothesized that energy stress in the tumour microenvironment serves as a key signal to stimulate GLUT3 expression in CRC cells to withstand nutrient scarcity and to exacerbate the malignancy of CRC cells. In this study, we tested this hypothesis and were able to determine a new CRC metabolic vulnerability with therapeutic potential.

## Results

### GLUT3 is highly expressed in CRC patient tissues and correlated with poor clinical outcomes

**Fig. 1:**
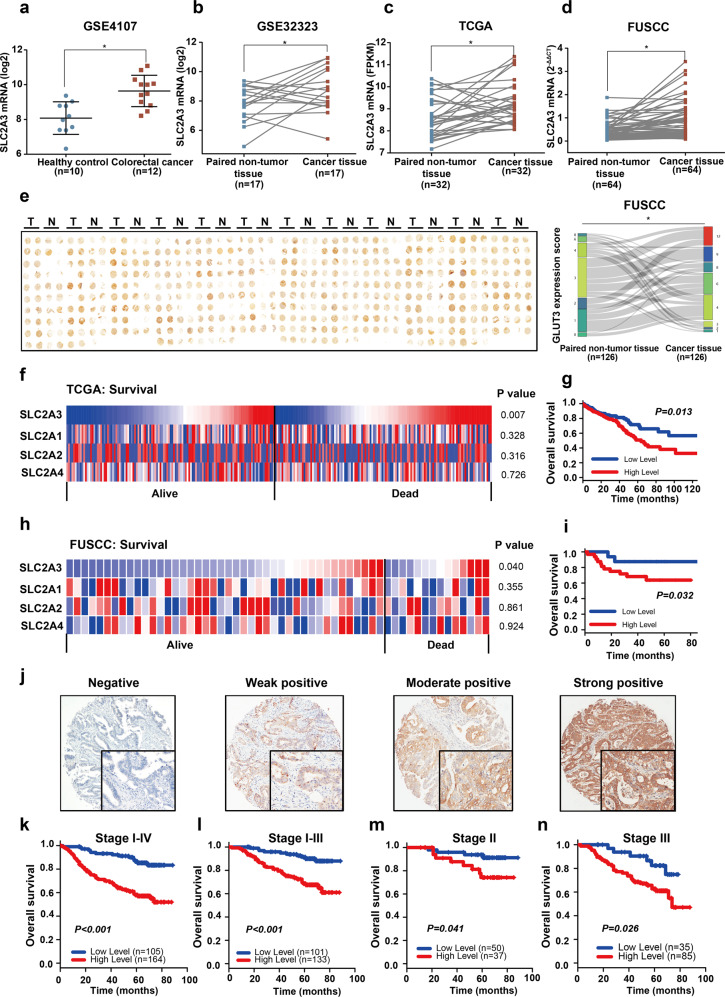
GLUT3 is highly expressed in CRC patient tissues and correlated with poor clinical outcomes. **a** The GEO dataset GSE4017 indicates *SLC2A3* expression levels in specimens from CRC patients and healthy donors. **b** The GEO dataset GSE32323 indicates *SLC2A3* expression levels in CRC tissues and paired adjacent normal tissues. **c** TCGA datasets indicate *SLC2A3* expression levels in paired adjacent normal tissues and tumour tissues from CRC patients. **d** *SLC2A1-4* expression between paired normal tissues and tumour tissues from patients derived from the FUSCC database. **e** Immunohistochemical staining of CRC tumour tissues and paired normal tissue microarrays from patients at the FUSCC using anti-GLUT3 antibody. **f** Difference in SLC2A1-4 expression between patients who died within three years and patients with long-term survival in TCGA database. **g** Kaplan–Meier analysis of SLC2A3 expression (median as the cut-off point) and overall survival in data from TCGA database. **h** Difference in SLC2A3 expression between patients who died within three years and patients with long-term survival in the FUSCC database. **i** Kaplan–Meier analysis of SLC2A3 expression (median as the cut-off point) and overall survival in data from TCGA database. **j** Representative immunohistochemical staining for GLUT3 protein expression in CRC patients with different intensity. **k**–**n** Kaplan–Meier analysis of GLUT3 expression and overall survival in the overall FUSCC cohort (**k**), patients with stage I-III CRC (**l**), patients with stage II CRC (**m**), and patients with stage III CRC (**n**)

To ascertain expression levels of the GLUT transporter isoforms GLUT1, GLUT2, GLUT3 and GLUT4 in neoplastic tissues of patients with CRC, we first performed bioinformatics analyses of the *SLC2A1*, *SLC2A2*, *SLC2A3* and *SLC2A4* mRNA levels using the public RNA-seq datasets from TCGA and RNA microarray datasets from GEO. Only *SLC2A3* was dramatically upregulated in the CRC tissues of patients compared to the colonic tissues of healthy volunteers (Fig. 1a, Supplementary Fig. S1a–c). In addition, comparison of data from matched adjacent benign colorectal tissues and CRC patient tissues from both GSE32323 and TCGA revealed higher *SLC2A3* mRNA levels, while only CRC tissue data from the TCGA showed increased *SLC2A1* mRNA levels (Fig. 1b, c and Supplementary Fig. S1d–i). To validate this finding, we enroled a patient cohort (cohort 1) containing 64 cases with CRC at the Fudan University Shanghai Cancer Center (FUSCC) and harvested their paired normal and CRC tissues to conduct q-PCR assay to detect GLUT isoform mRNAs. We found that the mRNA levels of both *SLC2A1* and *SLC2A3* were significantly upregulated in cancer tissues of these CRC patients (Fig. 1d, Supplementary Fig. S1j–l). Subsequently, we asked whether expression of GLUT1 and GLUT3 protein was upregulated in CRC tissues. Hence, we recruited another cohort (cohort 2) containing 269 cases with CRC. Notably, we collected paired CRC tumour and non-tumour tissues from 126 cases, while we only obtained CRC tumour tissues from the remaining 143 cases. All these specimens were used for tissue microarray preparation and subsequent immunohistochemical (IHC) assays. For the 126 pairs of CRC tumour and non-tumour tissues, both GLUT1 and GLUT3 were dramatically upregulated in CRC tissues as relative to benign tissues. The percentages of patients with positive GLUT3 expression or GLUT1 expression were 63.49% and 71.43% respectively (Fig. 1e and Supplementary Fig. S1m). Collectively, these data demonstrated that GLUT1 and GLUT3 were upregulated at both the transcription and translation levels in CRC tissues of patients.

Next, we asked if GLUT3 expression and the therapeutic outcomes of patients with CRC are associated. Notably, in addition to GLUT3, GLUT1, GLUT2 and GLUT4,^12^ which are well-studied glucose transporters in cancer cells, were analysed. Public TCGA data and the GSE17538 and GSE39582 datasets were used to assess the association between transcription of the genes encoding these transporters and the long-term overall survival of patients with CRC. Among these four genes, expression of only *SLC2A3* was significantly higher in patients who died within 3 years than in those who survived more than >3 years, and only this gene was a negative prognostic factor for long-term survival (>3 years) (Fig. 1f, Supplementary Fig. S1n, o). Furthermore, Kaplan–Meier analysis confirmed that *SLC2A3*, but not *SLC2A1, SLC2A2* or *SLC2A4*, was negatively correlated with poor long-term overall survival of patients in all three public datasets (Figs. 1g, S1p, q, S2a–i). Consistently, qPCR data from the FUSCC dataset validated this finding (Fig. 1h, i, Supplementary Fig. S2j–l). Notably, correlation analyses based on the FUSCC cohort suggested that only *SLC2A3* was highly expressed in specimens from tumours with poor differentiation (Supplementary Fig. S2m, n), specimens from mucinous-type tumours (Supplementary Fig. S2o, p) and specimens from right side tumours (Supplementary Fig. S2q, r). To determine the prognostic value of GLUT1 and GLUT3 protein expression levels, tumour samples from tissue microarray (*n* = 269) were used for IHC staining. The result revealed that high level of GLUT3 was closely associated with inferior overall survival of patients with distinct pathologic stages (Fig. 1j–n), while GLUT1 was not associated with prognosis of CRC (Supplementary Fig. S3a–c). The relationship between GLUT3 protein expression levels and clinicopathological features was further assessed and we found that high expression of GLUT3 was significantly associated with poor features including advanced tumour stage, poor differentiation and right sided cancer (Supplementary Table S1).

To ascertain whether GLUT3 had prognostic potential for other solid tumours, the association between *SLC2A3* mRNA levels and long-term survival was analysed in 16 solid tumours using TCGA datasets. We found that high levels of *SLC2A3* mRNA predicted unfavourable prognosis in the nine solid tumours: stomach, pancreatic, renal, head and neck, ovarian, thyroid, urothelial, cervical and glioma cancers (Supplementary Fig. S3D, Supplementary Table S2). Therefore, *SLC2A3* may be a potential prognostic biomarker for these tumours.

### GLUT3-mediated glucose utilization is essential for CRC growth in vitro and in vivo

**Fig. 2:**
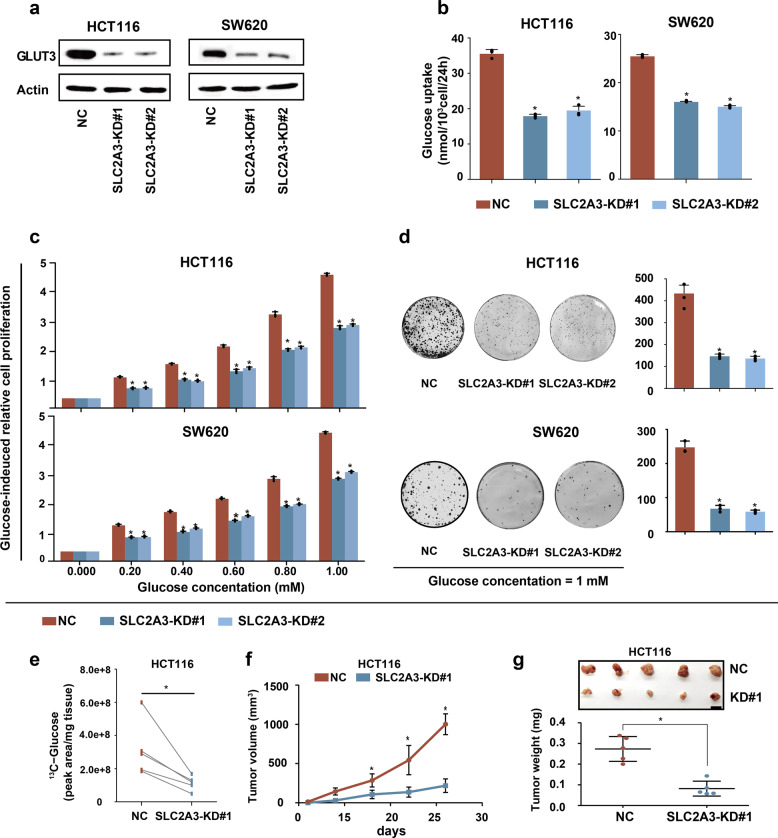
Glucose utilization mediated by GLUT3 is required to promote CRC growth in vitro and in vivo. **a** Western blot showing short hairpin RNA-mediated deletion of *SLC2A3* in HCT116 and SW620 cells. NC, nontarget control; KD, knockdown. **b** Glucose uptake by HCT116 and SW620 cells with or without *SLC2A3* silencing. **c** Glucose-induced proliferation of HCT116 and SW620 cells with or without *SLC2A3* depletion. **d** Analysis of colony formation abilities of HCT116 and SW620 cells with or without *SLC2A3* depletion. **e** ^13^C-glucose uptake by HCT116 xenografts with or without *SLC2A3* deletion. **f**, **g** Subcutaneous tumour growth, xenograft tumour images and tumour weights of xenografts from HCT116 cells with or without *SLC2A3* silencing in nude mice

To determine whether GLUT3-mediated glucose utilization is requisite for the rapid growth of CRC cells in vitro and in vivo, we silenced *SLC2A3* in two cell lines, HCT116 and SW620, which show high baseline expression of GLUT3, by using small short hairpin RNA (Fig. 2a; Supplementary Fig. S4a). In vitro, *SLC2A3* knockdown significantly suppressed glucose uptake (Fig. 2b), inhibited glucose-induced cell proliferation (Fig. 2c), attenuated colony formation ability (Fig. 2d) and induced cell cycle arrest (Supplementary Fig. S4b, c). In vivo, *SLC2A3* silencing strikingly reduced the ^13^C-glucose level of HCT116 xenografts (Fig. 2e). Consequently, these two cell lines with depressed glucose uptake caused by *SLC2A3* depletion developed significantly smaller and lighter tumours than control cells (Fig. 2f, g; Supplementary Fig. S4d–f). Moreover, IHC analysis of xenografts from the immunocompromised mice showed substantially decreased Ki67 (Supplementary Fig. S4g, h). Collectively, these results show that GLUT3-mediated glucose utilization is necessary for the rapid growth of CRC in vitro and in vivo.

**Fig. 3:**
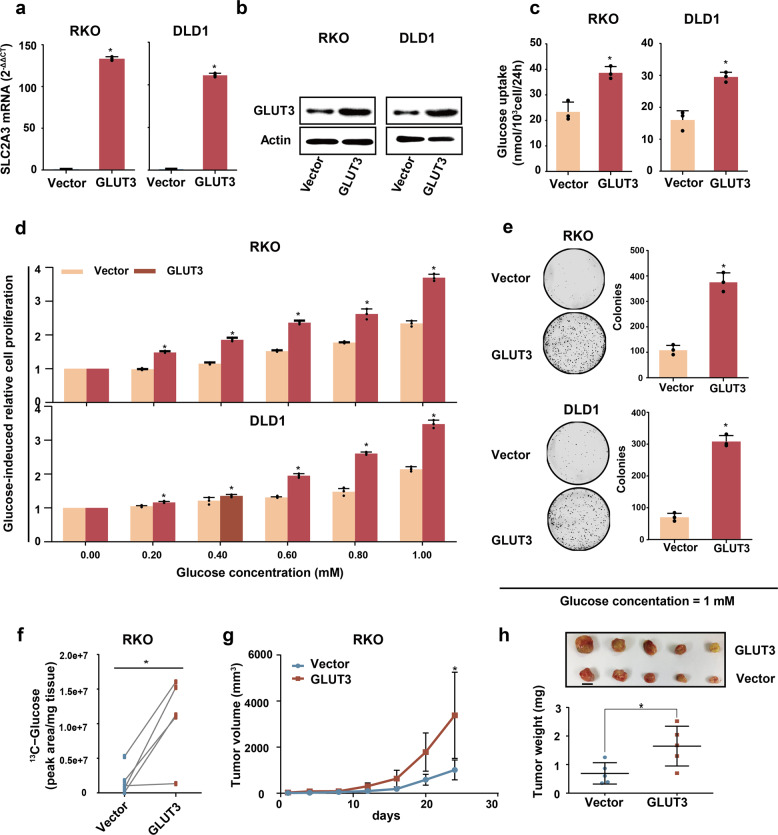
Enhanced fructose utilization mediated by GLUT3 promotes CRC growth in vitro and in vivo. **a**, **b** RT-PCR (**a**) and western blot analysis (**b**) showing SLC2A3 expression in RKO and DLD1 cells transfected with the control PCDH retrovirus or PCDH-GLUT3 retrovirus. **c** Glucose uptake by RKO and DLD1 cells with or without ectopic GLUT3 expression. **d** Glucose-induced proliferation of RKO and DLD1 cells with or without ectopic GLUT3 expression. **e** Analysis of the colony formation abilities of RKO and DLD1 cells with or without ectopic GLUT3 expression. **f** ^13^C-glucose uptake by RKO xenografts with or without ectopic GLUT3 expression. **g**, **h** Subcutaneous tumour growth, xenograft tumour images and tumour weights of xenografts from RKO cells with or without ectopic GLUT3 expression in nude mice

Since GLUT3 was remarkably upregulated in CRC tissues, we assumed that GLUT3 is used by CRC cells to accelerate glucose input and promote cell growth. To verify this hypothesis, the GLUT3 protein was overexpressed in two cell lines that show relatively low baseline expression of GLUT3, RKO and DLD1 cells (Fig. 3a, b). Compared with the control cells, CRC cells with ectopic GLUT3 expression exhibited increased rates of glucose uptake (Fig. 3c) and expedited glucose-induced cell proliferation (Fig. 3d), colony formation under low-glucose conditions (Fig. 3e) and accumulation of cells in the S phase of the cell cycle (Supplementary Fig. S4i). In vivo studies demonstrated that overexpression of the GLUT3 protein in RKO tumours significantly increased the intratumor ^13^C-glucose level (Fig. 3f). Moreover, cells with overexpression of GLUT3 exhibited accelerated subcutaneous tumour growth (Fig. 3g, h, Supplementary Fig. S4j, k) and upregulated Ki67 in vivo (Supplementary Fig. S4l, m). In summary, enhanced glucose utilization mediated by GLUT3 could accelerate the tumour growth of CRC cells in vitro and in vivo.

### GLUT3 had a greater impact on cell growth than GLUT1 under low-glucose conditions

**Fig. 4:**
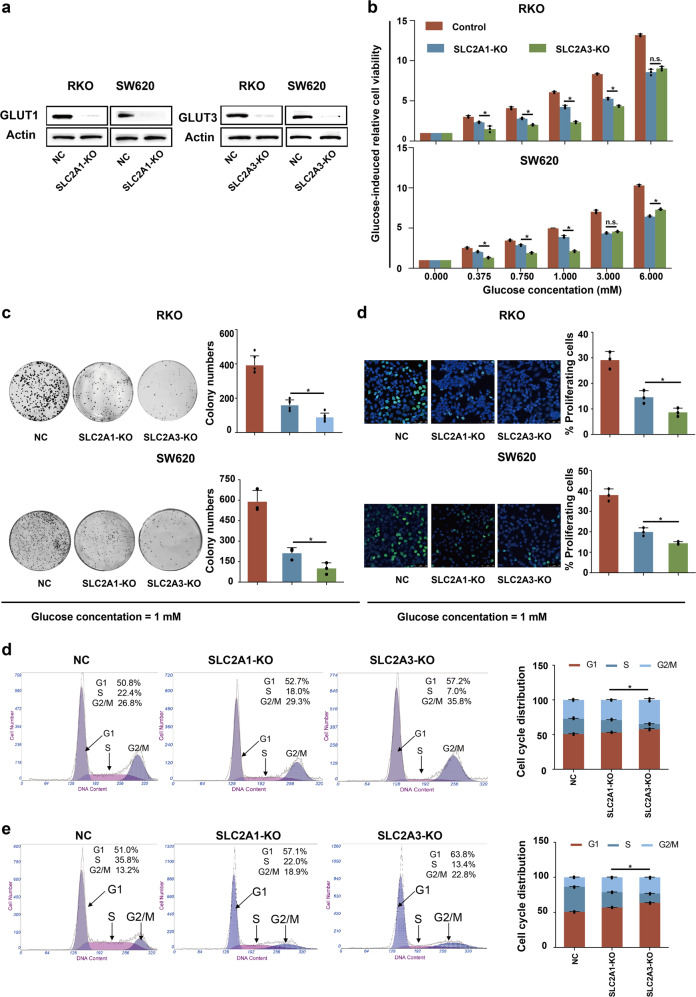
GLUT3 has higher impact than GLUT1 on CRC cell growth under low-glucose conditions. **a** Western blot showing CRISP-Cas9-mediated deletion of SLC2A1 and SLC2A3 in RKO and SW620 cells. NC, nontarget control; KO, knockout. **b** Glucose-induced proliferation of RKO and SW620 cells with/without SLC2A1 or SLC2A3 ablation. **c** Analysis of the colony formation abilities of RKO and SW620 cells with/without *SLC2A1* or *SLC2A3* ablation. **d** EdU staining for RKO and SW620 cells with/without *SLC2A1* or *SLC2A3* deletion. **e** Cell cycle distribution of RKO cells with/without *SLC2A1* or *SLC2A3* deletion. **f** Cell cycle distribution of SW620 cells with/without *SLC2A1* or *SLC2A3* deletion

In addition to GLUT3 reported here, GLUT1, a ubiquitous glucose transporter, was found to be upregulated in patient CRC tissues by our analysis and a previous study.^23^ We wondered whether GLUT3 has a unique function and thus cannot be substituted by GLUT1. Based on the higher affinity of GLUT3 compared to GLUT1 for glucose, we assumed that this transporter is indispensable for CRC cells to withstand glucose scarcity in the tumour microenvironment. To verify this assumption, a CRISPR-Cas9 approach was used to abrogate *SLC2A1* or *SLC2A3* in the RKO or SW620 CRC cell line, respectively (Fig. 4a). Deletion of either *SLC2A1* or *SLC2A3* significantly inhibited cell growth under different concentrations of glucose (Fig. 4b). However, when the glucose level was low (≤1 mM), CRC cells in which *SLC2A3* had been deleted showed a lower growth rate than CRC cells in which *SLC2A1* had been deleted (Fig. 4b), indicating that GLUT3 is more important than GLUT1 for CRC cell growth under conditions of glucose deficiency. In line with this finding, CRC cells with *SLC2A3* deletion showed attenuated colony formation (Fig. 4c), DNA replication (Fig. 4d) and enhanced cell cycle arrest (Fig. 4e) compared to that of CRC cells with *SLC2A1* deletion under conditions of 1 mM glucose. In summary, GLUT3 was more essential than GLUT1 for CRC cell growth under glucose-limiting conditions.

### In vivo glucose utilization mediated by GLUT3 uptake preferentially fuels nucleotide synthesis

**Fig. 5:**
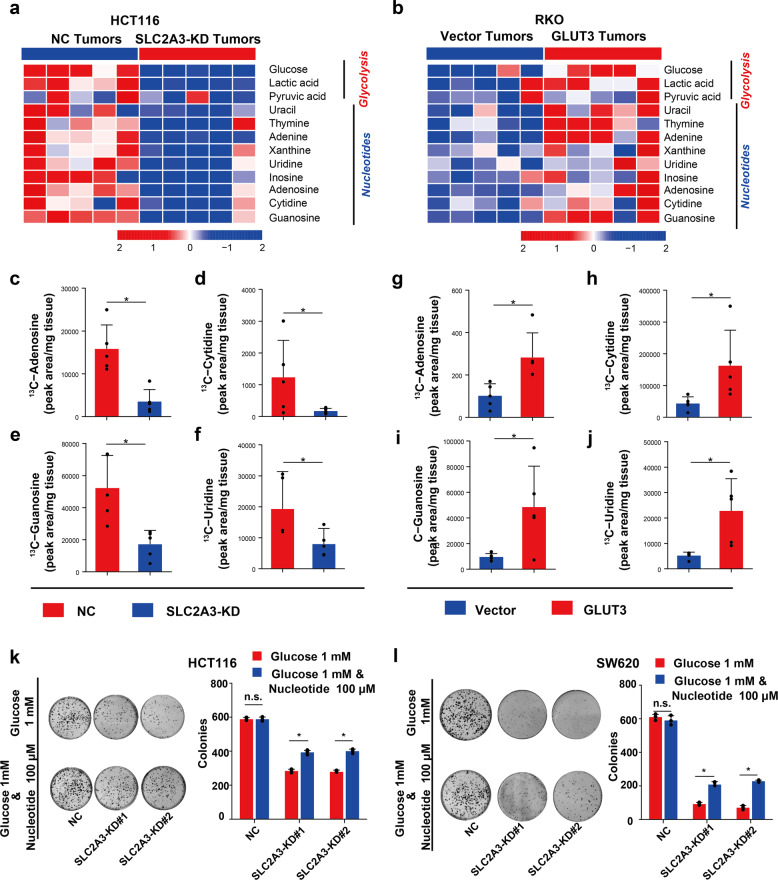
In vivo glucose utilization mediated by GLUT3 uptake preferentially fuels nucleotide synthesis. **a** The influence of impaired glucose utilization induced by *SLC2A3* silencing on glucose-derived metabolite production and the synthesis of nucleotides in HCT116 xenografts. **b** The impact of enhanced glucose utilization induced by GLUT3 overexpression on glucose-derived metabolite generation and the synthesis of nucleotides of RKO xenografts. **c**–**f** Production of ^13^C-labelled metabolites derived from 13C-glucose in HCT116 xenografts with or without *SLC2A3* silencing. **g**–**j** Generation of ^13^C-labelled metabolites derived from 13C-fructose in RKO xenografts with or without ectopic GLUT3 expression. **k**–**l** Analysis of the colony formation abilities of HCT116 (**k**) and SW620 (**l**) cells with or without *SLC2A3* silencing during nucleoside rescue

To elucidate the underlying metabolic mechanism responsible for GLUT3-induced tumour growth in vivo, we used a gas chromatography-time-of-flight mass spectrometry (GC-TOFMS)-based untargeted metabolomics approach to detect the impact of GLUT3-mediated glucose utilization on the global metabolism of CRC xenografts. Xenografts of HCT116 cells with or without *SLC2A3* silencing and xenografts of RKO cells with or without GLUT3 protein overexpression were used for this investigation. The metabolites influenced by GLUT3 expression are shown in Data S1. We found that *SLC2A3* knockdown in HCT116 tumours strikingly reduced the levels of glucose, pyruvic acid, lactate and many nucleotides, while ectopic expression of GLUT3 in RKO tumours significantly raised the levels of these metabolites (Fig. 5a, b). Based on these findings, we hypothesized that the glucose taken up by GLUT3 is preferentially catabolized to synthesize nucleotides and activate glycolysis in CRC.

To verify the above hypothesis, we conducted the following in vivo metabolic flux assays. We intravenously infused ^13^C-labelled glucose into xenograft tumour-bearing mice and traced the ^13^C-glucose-derived metabolites in xenografts. We observed that ^13^C-adenosine, ^13^C-cytidine, ^13^C-guanosine and ^13^C-uridine were dramatically reduced in HCT116 tumours with *SLC2A3* silencing (Fig. 5c–f), whereas these ^13^C-labelled metabolites were significantly upregulated in RKO tumours with forced GLUT3 expression (Fig. 5g–j).

To ascertain whether fuelling of these nucleotides by GLUT3-mediated glucose utilization is essential to promote CRC growth in vitro, we cultured HCT116 and SW620 cells with or without *SLC2A3* silencing in medium supplemented with a nucleotide mixture containing adenosine, cytidine, guanosine and uridine (100 μM). The colony formation of CRC cells with *SLC2A3* knockdown was significantly enhanced by nucleotide supplementation (Fig. 5k, l), and the growth inhibition and cell cycle arrest of CRC cells caused by *SLC2A3* silencing were significantly restored (Supplementary Fig. S5a–d). Taken together, these results show that glucose utilization mediated by GLUT3 promoted CRC cell growth by fuelling nucleotide synthesis.

### Glucose deficiency activates AMPK and induces GLUT3 expression in CRC cells via CREB1

**Fig. 6:**
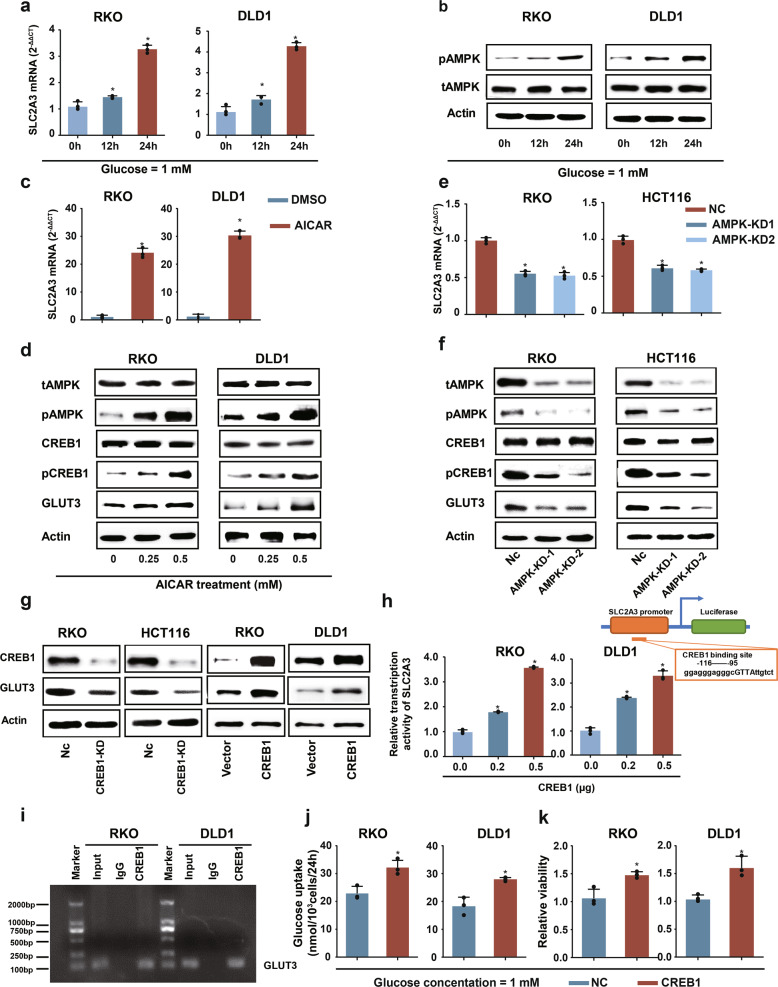
Glucose deficiency activates AMPK and induces GLUT3 expression in CRC cells via CREB1. **a** mRNA expression of *SLC2A3* in RKO and DLD1 cells cultured in 1-mM glucose-containing medium for 0–24 h. **b** Activation of AMPK in RKO and DLD1 cells cultured in 1-mM glucose-containing medium for 0–24 h. **c** mRNA expression of *SLC2A3* in RKO and DLD1 cells treated with or without the AMPK activator AICAR. **d** Western blot of CREB1, phosphorylated CREB1 and GLUT3 in RKO and DLD1 cells treated with or without the AMPK activator AICAR. **e** mRNA expression of *SLC2A3* in RKO and HCT116 cells transfected with or without AMPK siRNA. **f** Western blot of CREB1, phosphorylated CREB1 and GLUT3 in RKO and DLD1 cells treated with or without AMPK siRNA. **g** Expression of GLUT3 in RKO and HCT116 cells with CREB1 silencing and in RKO and DLD1 cells with enhanced CREB1 expression. **h** Ectopic CREB1 expression in RKO and DLD1 cells increased the transcriptional activity of the entire *SLC2A3* promoter. **i** ChIP results showing that CREB1 can occupy the genomic region of the *SLC2A3* promoter. **j** The influence of ectopic CREB1 expression on glucose uptake by RKO and DLD1 cells. **k** The influence of ectopic CREB1 expression on the proliferation of RKO and DLD1 cells cultured in complete medium containing 1-mM glucose for 48 h

An important remaining question was the mechanism of enhanced GLUT3 expression in CRC cells. In a previous study, we conducted a comprehensive metabolomics investigation of tissue samples from 193 patients with CRC and found that glucose levels were strikingly reduced in the CRC tissues of patients compared to adjacent non-tumour tissues.^27^ Glucose scarcity results in decreased energy production and activation of AMP-activated protein kinase (AMPK), an upstream regulator of many metabolic genes.^28,29^ Therefore, we hypothesized that glucose deficiency in the TME enhanced AMPK signalling to elicit GLUT3 expression. We detected alterations in *SLC2A3* transcription and AMPK activation under conditions of glucose deprivation. Glucose deprivation strikingly increased *SLC2A3* transcription and stimulated AMPK signalling over time (Fig. 6a, b; Supplementary Fig. S6a). To determine whether AMPK activation impacted the level of GLUT3, the AMPK activator AICAR and the AMPK inhibitor compound C were used to treat CRC cells. Activation of AMPK by AICAR significantly upregulated *SLC2A3* transcription (Fig. 6c) and GLUT3 production (Fig. 6d), while inhibition of AMPK by compound C had the opposite effect (Supplementary Fig. S6b, c). Furthermore, AMPK silencing using small interfering RNA significantly attenuated *SLC2A3* transcription and GLUT3 production (Fig. 6e, f).

Subsequently, we determined how glucose deficiency-stimulated AMPK modulated GLUT3 expression in CRC cells. We used MatInspector software to search for transcription factor binding sites and found a CREB1-binding site in the promoter region of *SLC2A3* (Supplementary Table S3). It was reported that CREB1 can be recognized and stimulated by AMPK by phosphorylating it at Ser133.^30^ In CRC cells, stimulation of AMPK activity by AICAR treatment activated CREB1 and upregulated GLUT3 expression (Fig. 6d), while repression of AMPK activity by compound C treatment or by downregulation of AMPK reduced phosphorylated CREB1 and downregulated GLUT3 (Fig. 6f and Supplementary Fig. S6c). To further confirm that CREB1 can modulate GLUT3 expression, we downregulated CREB expression or overexpressed CREB in CRC cells and observed the impact on *SLC2A3* transcription and GLUT3 expression. Downregulation of CREB1 decreased *SLC2A3* transcription and GLUT3 expression, whereas overexpression of CREB1 promoted *SLC2A3* transcription and GLUT3 expression (Supplementary Fig. S6d and 6g). In addition, we carried out a luciferase assay and confirmed that CREB1 could enhance *SLC2A3* promoter transcription in a dose-dependent manner (Fig. 6h). Furthermore, chromatin immunoprecipitation (ChIP)-qPCR analysis was performed, the results of which indicated that CREB could bind the promoter region of *SLC2A3* in RKO (Fig. 6i) and DLD1 (Fig. 6i) cells. Finally, we determined whether CREB1-induced GLUT3 had normal metabolic function. Concomitant with the upregulation of GLUT3 induced by CREB1 overexpression (Fig. 6g), glucose uptake and glucose-induced cell proliferation were enhanced in RKO and DLD1 cells (Fig. 6j, k). In contrast, when GLUT3 expression was reduced by CREB1 downregulation (Fig. 6f) in RKO and HCT116 cells, glucose uptake and glucose-induced cell proliferation were restrained (Supplementary Fig. S6e, f). Collectively, these findings show that glucose scarcity induced GLUT3 expression in CRC cells by activating the AMPK-CREB1 signalling pathway and consequently enhanced the capability of these cells to increase their intake of glucose and thus withstand low-glucose stress.

Notably, we confirmed that high CREB1 expression predicts the unfavourable overall survival of patients with CRC (Supplementary Fig. S6g). In addition, the positive correlation between GLUT3 and CREB1 in clinical CRC specimens was verified by IHC staining analysis (Supplementary Fig. S6h, i).

### High expression of GLUT3 in CRC provides a therapeutic opportunity

Since clinical CRC tissues displayed dramatically elevated GLUT3 expression compared to that in matched adjacent normal tissues, we explored potential therapeutic targets generated by the CRC-induced expression of this transporter. Both GLUT1 and GLUT3 can import the oxidized form of vitamin C, dehydroascorbate (DHA), and intracellular DHA can be rapidly reduced to vitamin C by the oxidation of glutathione (GSH), thioredoxin, and nicotinamide adenine dinucleotide phosphate (NADPH), thereby causing severe oxidative stress, inactivation of glyceraldehyde 3-phosphate dehydrogenase, blockage of glycolysis and ultimately, cell death.^31^ Of note, intracellular vitamin C can inhibit GLUT3 and block glucose uptake mediated by this transporter.^32^ Therefore, we assumed that high expression of GLUT3 in CRC tissues could be used to enhance cellular DHA uptake, resulting in the depletion of intracellular antioxidants, energetic crisis and cell death. To test the effect of vitamin C mediated by GLUT3 on redox homeostasis and glycolytic activity, an in vitro vitamin C treatment assay was conducted using CRC cells with or without forced expression of GLUT3. Vitamin C in cell culture medium can be oxidized to DHA with a half-life of 70 min.^31^ After vitamin C treatment for 24 h, relative to control cells, CRC cells with ectopic GLUT3 expression exhibited an obvious increase in intracellular ROS levels (Supplementary Fig. S7a) and a severe decrease in intracellular GSH levels (Supplementary Fig. S7b). Accompanied by the impairment of intracellular reducing capability, the production of lactate and ATP was significantly impeded in CRC cells with GLUT3 overexpression relative to control cells under treatment with vitamin C (Supplementary Fig. S7c, d). Therefore, high GLUT3 expression in CRC cells and its subsequent ability to increase DHA uptake could be exploited to elevate intracellular oxidative stress and cause cell death.

**Fig. 7:**
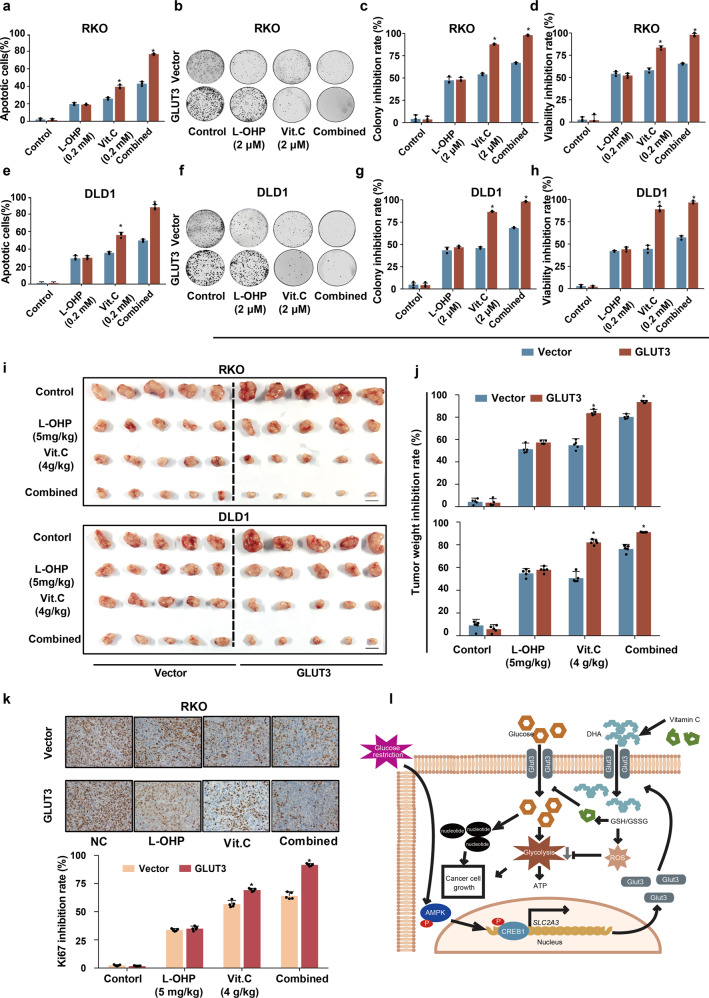
High GLUT3 expression in CRC provides a therapeutic opportunity. **a** Analysis of the apoptosis of RKO cells with or with ectopic GLUT3 expression treated with L-OHP alone, vitamin C alone or the two agents combined. **b**, **c** Analysis of the colony formation ability of RKO cells with or with ectopic GLUT3 expression treated with L-OHP alone, vitamin C alone or the two agents combined. **d** Analysis of cell proliferation inhibition in RKO cells with or with ectopic GLUT3 expression treated with L-OHP alone, vitamin C alone or the two agents combined. **e** Analysis of the apoptosis of DLD1 cells with or with ectopic GLUT3 expression treated with L-OHP alone, vitamin C alone or the two agents combined. **f**, **g** Analysis of the colony formation ability of DLD1 cells with or with ectopic GLUT3 expression treated with L-OHP alone, vitamin C alone or the two agents combined. **h** Analysis of cell proliferation inhibition in DLD1 cells with or with ectopic GLUT3 expression treated with L-OHP alone, vitamin C alone or the two agents combined. **i**, **j** In vivo analysis of the treatment effects of L-OHP alone, vitamin C alone and the two agents combined on xenografts from RKO and DLD1 cells with or without enhanced GLUT3 expression. **k** Immunohistochemical staining for Ki67 in xenografts from RKO cells with or without enhanced GLUT3 expression in nude mice treated with normal saline, L-OHP alone, vitamin C alone or the two agents combined

To further investigate the therapeutic value of vitamin C for the destruction of CRC cells with high GLUT3 expression, we tested the impact of vitamin C alone as well as in combination with the common anticancer agent oxaliplatin (L-OHP) on CRC cell growth. Forced expression of GLUT3 significantly increased the sensitivity of CRC cells to vitamin C, as shown by the higher percentage of apoptotic cells and increased inhibition of colony formation relative to those of control cells (Fig. 7a–h). Overexpression of GLUT3 did not alter the sensitivity of CRC cells to L-OHP (Fig. 7a–h). However, forced expression of GLUT3 clearly promoted the synergistic effect of vitamin C and L-OHP in CRC cells (Fig. 7a–h). We then performed an in vivo study to evaluate the efficacy of the administration of vitamin C alone and in combination with L-OHP on CRC xenograft growth. In line with the in vitro findings, forced expression of GLUT3 markedly raised the sensitivity of CRC xenografts to vitamin C administration, as demonstrated by the increased inhibitory effect on tumour weight relative to that in control xenografts (Fig. 7i, j). In addition, ectopic expression of GLUT3 remarkably enhanced the synergistic effect of vitamin C and L-OHP in repressing CRC xenograft growth (Fig. 7i, j). An IHC assay to detect the cell proliferation marker Ki67 confirmed that high GLUT3 expression increased the sensitivity of CRC xenografts to the administration of vitamin C alone and in combination with L-OHP (Fig. 7k and Supplementary Fig. S7e). Notably, the average body weight of tumour-bearing mice treated with vitamin C or a combination of vitamin C and L-OHP was not significantly perturbed compared to that of the vehicle group, indicating the safety of the vitamin C and vitamin C-containing regimens (Supplementary Fig. S7f). In conclusion, high expression of GLUT3 in CRC cells could improve the therapeutic efficacy of the use of vitamin C alone and in combination with L-OHP, providing a new therapeutic possibility for this type of malignancy.

## Discussion

The pathological stage is a crucial clinical parameter for determining the treatment protocol of patients with CRC. Surgery is the major treatment protocol for stages I and II CRC, which exhibit recurrence rates of ~10% and 20%, respectively.^33^ Patients with stage III CRC usually receive surgery and adjuvant chemotherapy and exhibit a recurrence rate of more than 50%.^2,33^ Palliative chemotherapy is the only option for patients with distant metastases (stage IV), who account for ~20% of patients with newly diagnosed CRC.^4^ New therapeutic substances, such as bevacizumab, which inhibits vascular endothelial growth factor (VEGF), and cetuximab, which acts by repressing epidermal growth factor receptor (EGFR), have been developed to treat CRC.^2^ In summary, new drug targets and therapeutic agents are still required to improve the treatment outcomes of patients with a high risk for distant metastases.

Several studies in recent years have unveiled the crucial role of metabolic reprogramming in tumorigenesis and tumour progression, yielding a series of new therapeutic targets and strategies.^34–37^ Our previous metabolomics study of clinical CRC tissue samples revealed glucose scarcity in the TME.^27^ In the current study, we demonstrated that low-glucose conditions serve as an upstream signal to stimulate the AMPK/CREB1 signalling pathway in CRC cells, subsequently upregulating GLUT3, a transmembrane transporter with a high affinity for glucose. CREB1 is a well-characterized transcription factor of the basic leucine zipper family. In response to various stimuli that elevate intracellular cAMP or Ca^2+^ levels, CREB is activated through phosphorylation at Ser133.^38^ The activation of CREB1 turns on the transcription of down-stream genes. As a downstream target of AMPK, CREB1 can be directly phosphorylated by AMPK, thus stimulating *SLC2A3* transcription. Another upstream stimulus that occurs in parallel with glucose deficiency in the CRC TME to boost GLUT3 expression is hypoxia.^39^ The individual contributions of hypoxia and glucose deficiency to the upregulation of GLUT3 in CRC cells have yet to be determined. Mechanistic experiments in this study showed that GLUT3 accelerated glucose uptake and that imported glucose was preferentially utilized to fuel nucleotide synthesis to expedite CRC cell growth. The collective results in cell lines in which AMPK or CREB1 was silenced or overexpressed and the effects of these manipulations on GLUT3 expression, identified the AMPK/CREB1/GLUT3 axis as a new alternative pathway to retain the metabolic activity of CRC cells in the face of glucose insufficiency.

As a major energy resource for cancer cells, glucose metabolism contains three main pathways in the cytoplasm including glycolysis pathway, pentose phosphate pathway (PPP), and serine synthesis pathway (SSP).^40^ PPP mainly contributes to the production of ribose-5-phosphate, which is an important precursor for many macromolecules, including nucleotides. However, our results revealed that glucose transported by GLUT3 was preferentially catabolized to synthesize nucleotides in CRC. Previous studies have revealed that GLUT3 is able to regulate the activities of several important signal pathways and then alter the expression of target genes in cancer.^41^ Hence, we hypothesize that GLUT3 may be able to regulate the expression of critical enzymes involved in nucleotide synthesis by targeting particular signal pathways. Of note, future studies should be conducted to elucidate the underlying mechanism of GLUT3 preferentially fuelling nucleotide synthesis.

Intriguingly, in this study, GLUT3 was found to play a role distinct from that of GLUT1 in CRC. First, although both *SLC2A1* and *SLC2A3* were upregulated in CRC tissues, the level of only *SLC2A3* was significantly and negatively associated with the overall survival of patients; the *SLC2A1* level was not predictive of overall CRC patient survival. Second, under low-glucose conditions, GLUT3 was more important than GLUT1 for CRC cell growth. These results indicated that GLUT3 expression may have both prognostic value and therapeutic potential. To thoroughly elucidate the unique role of each glucose transporter in CRC and other cancers, more studies will be required.

The most significant result of this study is the finding that high GLUT3 expression in CRC tissues yields a valuable therapeutic opportunity based on previous studies of the effect of vitamin C on GLUT3 and CRC cells.^31,32^ Vitamin C and overexpression of GLUT3 were used in this study to establish a cycle of CRC cell suicide. DHA transformed from vitamin C in the cell medium can be readily transported into cells by GLUT3, intracellular DHA is rapidly reduced to vitamin C by the consumption of abundant antioxidants, and intracellular vitamin C can inhibit GLUT3 and attenuate its ability to take up glucose. This suicide cycle generates not only high levels of oxidative stress, which is harmful for CRC cells, but also an energetic crisis due to the blockade of glycolysis and reduced GLUT3-mediated glucose input. Therefore, CRC cells with high GLUT3 expression were found to be highly sensitive to treatment with vitamin C or a vitamin C-containing regimen. DHA and the anticancer drug L-OHP worked in synergy to kill CRC cells. The data from this study indicated the considerable therapeutic benefit of vitamin C/DHA administration or vitamin C/DHA-containing regimens in CRC with high GLUT3 expression.

## Materials and methods

### Contact information for reagent and resource sharing

Detailed information regarding the resources used in this study is listed in the resource table. Further information and requests for resources and reagents should be directed to the lead contact, Guoxiang Cai (gxcai@fudan.edu.cn), who will respond to those requests.

### Human CRC samples

Two patient cohorts were recruited into the study at the Fudan University Shanghai Cancer Center (FUSCC). In cohort 1 (*n* = 64), a total of 64 pairs of benign colorectal tissues and cancer tissues was used to conduct mRNA expression analysis. In cohort 2 (*n* = 269), we collected cancer tissues and matched benign colorectal tissues from 126 cases, while we only obtained cancer tissues from the remaining 143 cases. These specimens were used for tissue microarray preparation and subsequent IHC staining. All participants provided informed written consent in accordance with the regulations of the Institutional Review Boards of the FUSCC.

### Human cell lines

The human CRC cell lines RKO, SW620, SW480, LoVo, HCT116, HCT8, HT29 and DLD1 were obtained from the National Cancer Institute (NCI). Human 293T cells (CRL-3214) were purchased from American Type Culture Collection (ATCC). All cell lines were maintained in DMEM supplemented with 10% foetal bovine serum (FBS, Thermo Fisher Scientific).

### Mice

We purchased 6- to 8-week-old female BALB/c-nude mice from the Shanghai Laboratory Animal Co., Ltd. (SLAC). Mouse studies were performed in specific pathogen-free (SPF) facilities with approval of the Institutional Animal Care and Use Committee of Fudan University. A subcutaneous xenograft mouse model was used in this study.

### Metabolomic profiling of tissue samples

A combination of gas chromatography-time-of-flight mass spectrometry (GC-TOFMS, LECO Corp., St Joseph, MI) and ultra-performance liquid chromatography-tandem mass spectrometry (UPLC-MS/MS, Waters Corp., Milford, MA) was used to measure small-molecule metabolites in the tissue samples. Metabolomics assays were conducted by Metabo-Profile, Inc. (Shanghai, China) using previously published methods.^27^ The metabolites were identified by comparison with an internal library built with standard reference chemicals.

### Glucose uptake assay

A total of 1 × 10^4^ CRC cells were cultured in 96-well plates containing glucose-free DMEM (Thermo Fisher Scientific) with 10% dialyzed foetal bovine serum (dFBS, Thermo Fisher Scientific) and 6 mM glucose, transferred to a CO_2_ incubator set at 37 °C and 5% CO_2_, and incubated for 48 h. Spent media were collected and used to measure the remaining fructose using a glucose colorimetric/fluorometric assay kit (Abcam) following the manufacturer’s instructions.

### Lentivirus transduction

GLUT3 shRNAs (TRCN0000043615 and TRCN0000042880) were purchased from Open Biosystems. Lentiviral vector for GLUT3 overexpression (pCDH-CMV-SLC2A3) was purchased from RiboBio (Guangzhou, China). Empty lentiviral vector served as the control. pCDH-CMV-SLC2A3 was mixed with the packaging plasmids psPAX2 and PMD2.G. These plasmids were then cotransfected into HEK293T cells using Lipofectamine 3000 reagent (Thermo Fisher Scientific) according to the manufacturer’s protocol. Viral particles were harvested at 48 h after transfection. Transduction with lentiviral particles was performed using polybrene (2 mg/ml, Sigma-Aldrich), and positive cells were selected with puromycin (Sigma-Aldrich).

### Subcutaneous tumour studies and xenograft preparation for metabolomic profiling

HCT116-NC and HCT116-SLC2A3-KO cells or SW620-NC and SW620-SLC2A3-KO cells were subcutaneously injected into the left and right hind flanks of 8-week-old female BALB/c-nude mice, respectively. Similarly, RKO/DLD1-vector and RKO/DLD1-GLUT5 cells were subcutaneously injected into the left and right hind flanks of 8-week-old female BALB/c-nude mice, respectively. The tumour length and width were measured every 3 or 4 days using a calliper. Tumour sizes were calculated as 0.5 × length × width^2^. At the end of the experiment, tumour xenografts were resected for imaging and weighing. Subsequently, these tumour xenografts were flash-frozen and stored in liquid nitrogen until the metabolomic assay.

### Tracer studies in mouse xenografts of CRC cells

We performed ^13^C-labelled tracer studies using a previously reported protocol with minor modifications. For tracer studies in mouse xenografts, on the final day of the experiment, the tumour-bearing mice were injected with 100 μL of 1 M [U-13C6]-D-fructose (Cambridge Isotope Laboratories) via the tail vein three times at 15-min intervals. One hour later, tumour xenografts were excised, weighed and flash-frozen in liquid nitrogen.

### Nucleoside rescue experiment

CRC cells were cultured in glucose-free DMEM with 10% dFBS, 6 mM fructose and 100 μM nucleosides (EmbryoMax nucleosides: Millipore) to observe the rescue efficacy for cells with GLUT3 deletion and attenuated glucose utilization.

### Analysis of the SLC2A3 promoter transcription activity

The entire *SLC2A3* promoter region was cloned and inserted into the luciferase promoter reporter vector pGL3-Basic. A CREB1 expression vector was constructed by inserting the full-length human CREB1 cDNA sequence into the pCDH-CMV-MCS-EF1-Puro vector (System Biosciences). The impact of CREB1 expression on the transcriptional activity of the *slc2a3* promoter was assessed in RKO and DLD1 cells by cotransfection of the CREB1 vector, luciferase promoter reporter vectors containing *slc2a3* promoter sequence, and the Renilla luciferase reporter vector pRL-SV40 (Promega). The luciferase activity was detected by using the Dual-Luciferase Reporter System (Promega).

### Chromatin immunoprecipitation (ChIP) assay

The CBEB1-binding site of the *SLC2A3* promoter was identified by using the online tool MatInspector (http://www.genomatix.de/matinspector. html). A ChIP assay to assess the binding of CREB1 to the *SLC2A3* promoter was conducted according to the manual provided by the ChIP Chromatin Immunoprecipitation Kit (Merck Millipore Corporation, Darmstadt, Germany).

### Measurement of cell apoptosis and analyses of the ROS signal and GSH/GSSG

According to the manufacturer’s instructions, a FITC Annexin V Apoptosis Detection Kit (BD Biosciences, La Jolla, CA, USA) was used to detect the apoptosis rate. The intracellular ROS level was detected by DCF-DA assay using the Cellular ROS Assay Kit (Abcam). The intracellular levels of GSH were measured using a GSH-Glo™ kit (Promega, Madison, WI, USA).

### EdU incorporation assay

5-Ethynyl-2′-deoxyuridine (EdU) is an analogue of thymidine, which is incorporated into proliferating cells during DNA synthesis. Therefore, a higher positive rate of EdU incorporation indicate a stronger cell proliferation ability. The EdU assay was performed by using an EdU detection kit (Beyotime Biotechnology, Shanghai, China) according to the manufacturer’s instruction.

### Analyses of lactate production and ATP levels

Cellular lactate production and ATP levels were measured using a Fluorometric Lactate Assay Kit (Abcam) and a Luminescent ATP Detection Assay Kit (Abcam), respectively.

### Treatment of CRC cell mouse xenografts with oxaliplatin and ascorbate acids

Oxaliplatin and ascorbate acid were dissolved in a dextrose solution and intraperitoneally injected into CRC-bearing mice at doses of 5 mg/kg and 4 g/kg at day 10 after subcutaneous transplantation of CRC cells. Oxaliplatin was administered twice per week, and ascorbate acid was administered twice a day. The vehicle group was administered the same volume of a dextrose solution.

## Supplementary information

Supplementary Information

Supplementary Table S1

## Data Availability

Source data and reagents are available from the corresponding author upon reasonable request.
