# Supplementary material for: GLUT3 induced by AMPK/CREB1 axis is key for withstanding energy stress and augments the efficacy of current colorectal cancer therapies
Source: Signal Transduct Target Ther. 2020 Sep 2;5:177. doi: 10.1038/s41392-020-00220-9 (PMC7463260; doi:10.1038/s41392-020-00220-9)
Supplement: Supplementary file 1 — Supplementary Information [file 41392_2020_220_MOESM1_ESM.docx]

Supplementary Materials for

**GLUT3 induced by AMPK/CREB1 axis is key for withstanding energy stress and augments the efficacy of current colorectal cancer therapies**

Weixing Dai^1,2†^, Ye Xu^1,2†^, Shaobo Mo^1,2†^, Qingguo Li^1,2†^, Jun Yu^3†^, Renjie Wang^1,2^, Yanlei Ma^1,2^, Yan Ni^8^, Wenqiang Xiang^1,2^, Lingyu Han^1,2^, Long Zhang^1,2^, Sanjun Cai^1,2^, Jun Qin^4*^, Wenlian Chen^5*^, Wei Jia^6,7*^, Guoxiang Cai^1,2*^

^1^ Department of Colorectal Surgery, Fudan University Shanghai Cancer Center

^2^ Department of Oncology, Shanghai Medical College, Fudan University, Shanghai, China, Shanghai

^3^ Department of Surgery, Johns Hopkins University School of Medicine, Baltimore, Maryland 21287, U.S.A.

^4^ The Key Laboratory of Stem Cell Biology, CAS Center for Excellence in Molecular Cell Science, Institute of Health Sciences, Shanghai Institutes for Biological Sciences, Chinese Academy of Sciences

^5^ Cancer Institute, Longhua Hospital, Shanghai University of Traditional Chinese Medicine, Shanghai 200032, China

^6^ University of Hawaii Cancer Center, Honolulu, HI 96813, USA

^7^ School of Chinese Medicine, Hong Kong Baptist University, Kowloon Tong, Hong Kong, China

^8^ The Children's Hospital, School of Medicine, Zhejiang University

†These authors contributed equally to this work

**Correspondence:**

Guoxiang Cai (E-mail: [gxcai@fudan.edu.cn](mailto:gxcai@fudan.edu.cn))

Wei Jia (Email: [wjia@cc.hawaii.edu](mailto:wjia@cc.hawaii.edu))

Wenlian Chen (Email: [CHENWL8412@shutcm.edu.cn](mailto:CHENWL8412@shutcm.edu.cn))

Jun Qin (Email: [qinjun@sibs.ac.cn](mailto:qinjun@sibs.ac.cn))

**This PDF file includes:**

Materials and Methods

Figures. S1 to S7

Tables S1 to S4

**Other Supplementary Materials for this manuscript include the following:**

Data S1

Materials and Methods

**Transfection**

siRNAs were purchased from GenePharma (Shanghai, China). Transfections of siRNAs and plasmids were performed using Lipofectamine 3000 (Thermo Fisher Scientific), according to the manufacturer’s protocol.

**Western blotting**

Western blotting was performed using whole-cell protein lysates of CRC cells using primary antibodies and a secondary antibody (anti-rabbit IgG, 1:7500; Cell Signaling Technology). Equal loading of protein samples was monitored using an anti-β-actin antibody (ab8226, 1:2500; Abcam).

**RNA isolation and quantitative real-time reverse transcription polymerase chain reaction (qRT-PCR)**

TRIzol reagent (Invitrogen, Carlsbad, CA, USA) was used to isolate total RNA and PrimeScript RT reagent (TaKaRa, Dalian, China) was used to obtain samples. The expression status of specific genes and β-actin were determined by qRT-PCR using an ABI 7900HT Real-Time PCR System (Applied Biosystems, Frederick, MD, USA). All reactions were run in triplicate.

**Immunohistochemistry (IHC) and histologic evaluation**

IHC was conducted as previously described (Li, Y. *et al. Molecular cancer.2016*). The immunohistochemically stained tissue sections were scored separately by two pathologists blinded to the clinicopathological parameters. The staining intensity was scored as 0 (negative), 1 (weak), 2 (medium) or 3 (strong). Extent of staining was scored as 0 (<5 %), 1(5–25 %), 2 (26–50 %), 3 (51–75 %) and 4 (>75 %) according to the percentages of the positive staining areas in relation to the whole carcinoma area. Scores for staining intensity and percentage positivity of cells were then multiplied to generate the immunoreactivity score (IRS) for each case. Samples having a final staining score of ≤ 4 were considered to be low and those with score of > 4 were considered to be high.


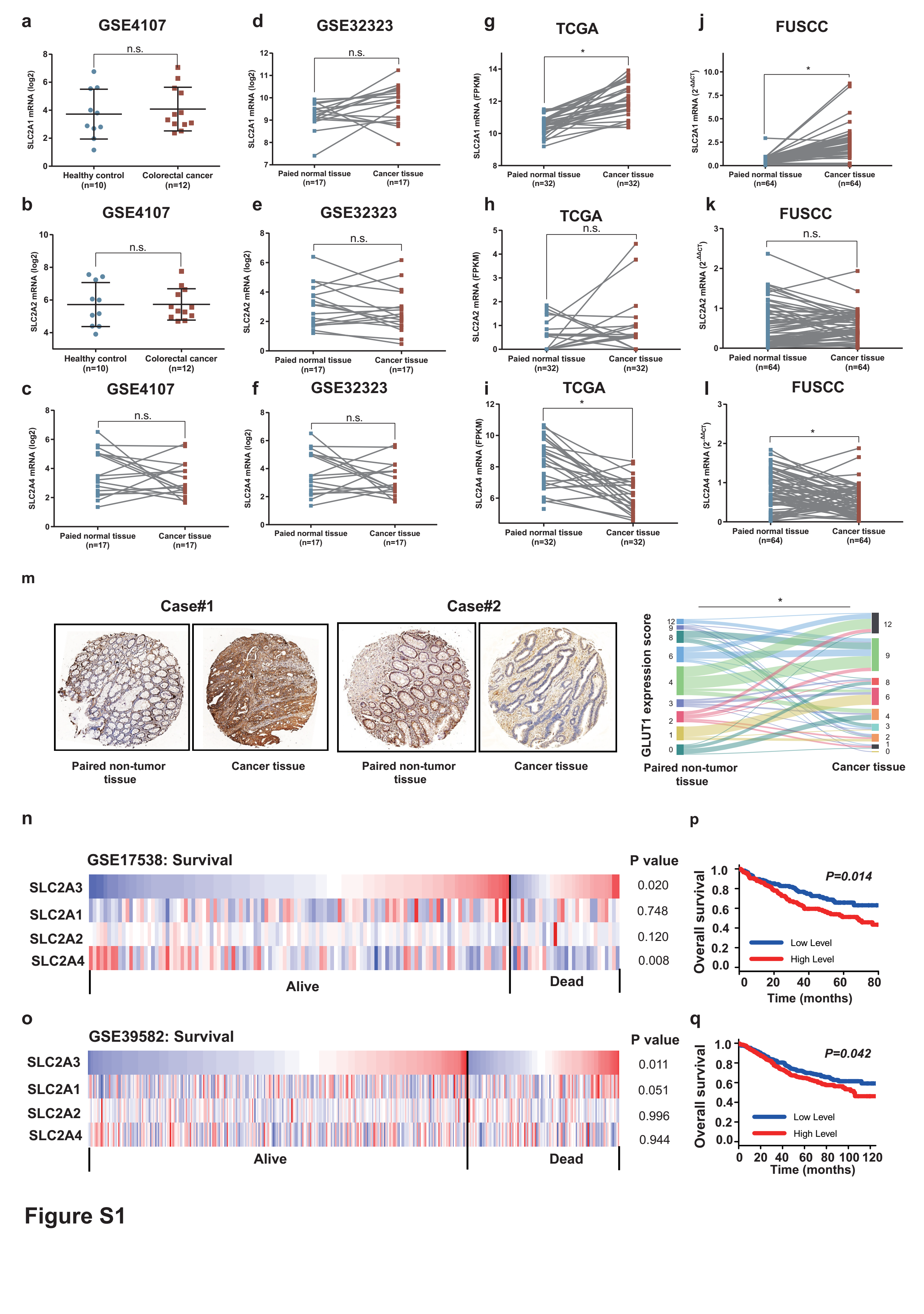
Figure. S1.

**Figure S1. Related to Figure 1. GLUT3 expression is significantly increased in CRC tissue, and specimens with short long-term survival.**

(a-c) GEO dataset GSE4017 showing SLC2A1, SLC2A2 and SLC2A4 expression between CRC and healthy specimens.

(d-f) GEO dataset GSE32323 showing SLC2A1, SLC2A2 and SLC2A4 expression between CRC tissues and paired adjacent normal tissues.

(g-i) TCGA datasets showing SLC2A1, SLC2A2 and SLC2A4 expression between paired adjacent normal tissues and tumor tissues from CRC patients.

(j-l) SLC2A1, SLC2A2 and SLC2A4 expression between paired normal tissues and tumor tissues in patients from FUSCC database.

(m) Immunohistochemical staining of CRC tumour tissues and paired normal tissue microarrays from patients at the FUSCC using anti-GLUT1 antibody.

(n-o) SLC2A1-4 expression between patients dead within three years and patients with long term survival in GSE17538 and GSE39582 database.

(p-q) Kaplan-Meier analysis between SLC2A3 expression (median as cutoff point) and overall survival in GSE17538 and GSE39582 database.


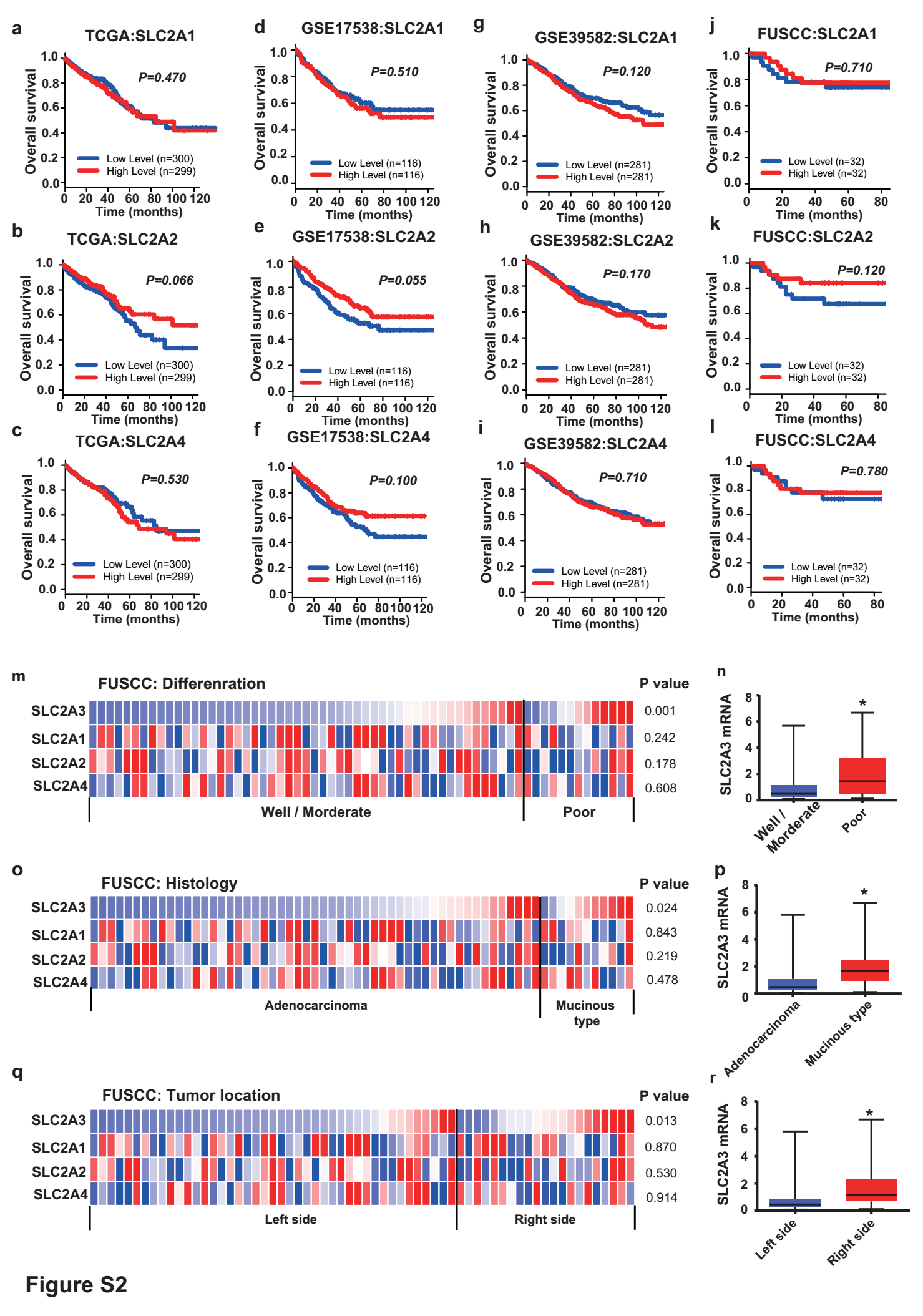
Figure. S2.

**Figure S2. Related to Figure 1. GLUT3 expression is significantly correlated to poor features and clinical outcomes.**

(a-c) Kaplan-Meier analysis between SLC2A1, SLC2A2, SLC2A4 expression (median as cutoff point) and overall survival in TCGA database.

(d-f) Kaplan-Meier analysis between SLC2A1, SLC2A2, SLC2A4 expression (median as cutoff point) and overall survival in GSE17538 database.

(g-i) Kaplan-Meier analysis between SLC2A1, SLC2A2, SLC2A4 expression (median as cutoff point) and overall survival in GSE39582 database.

(j-l) Kaplan-Meier analysis between SLC2A1, SLC2A2, SLC2A4 expression (median as cutoff point) and overall survival in FUSCC database.

(m-n) SLC2A1-4 expression between patients with poor differentiation and patients with well or moderate differentiation in FUSCC database.

(o-p) SLC2A1-4 expression between patients with mucinous histology and patients with adenocarcinoma in FUSCC database.

(q-r) SLC2A1-4 expression between right side CRC patients and left side CRC patients in FUSCC database.


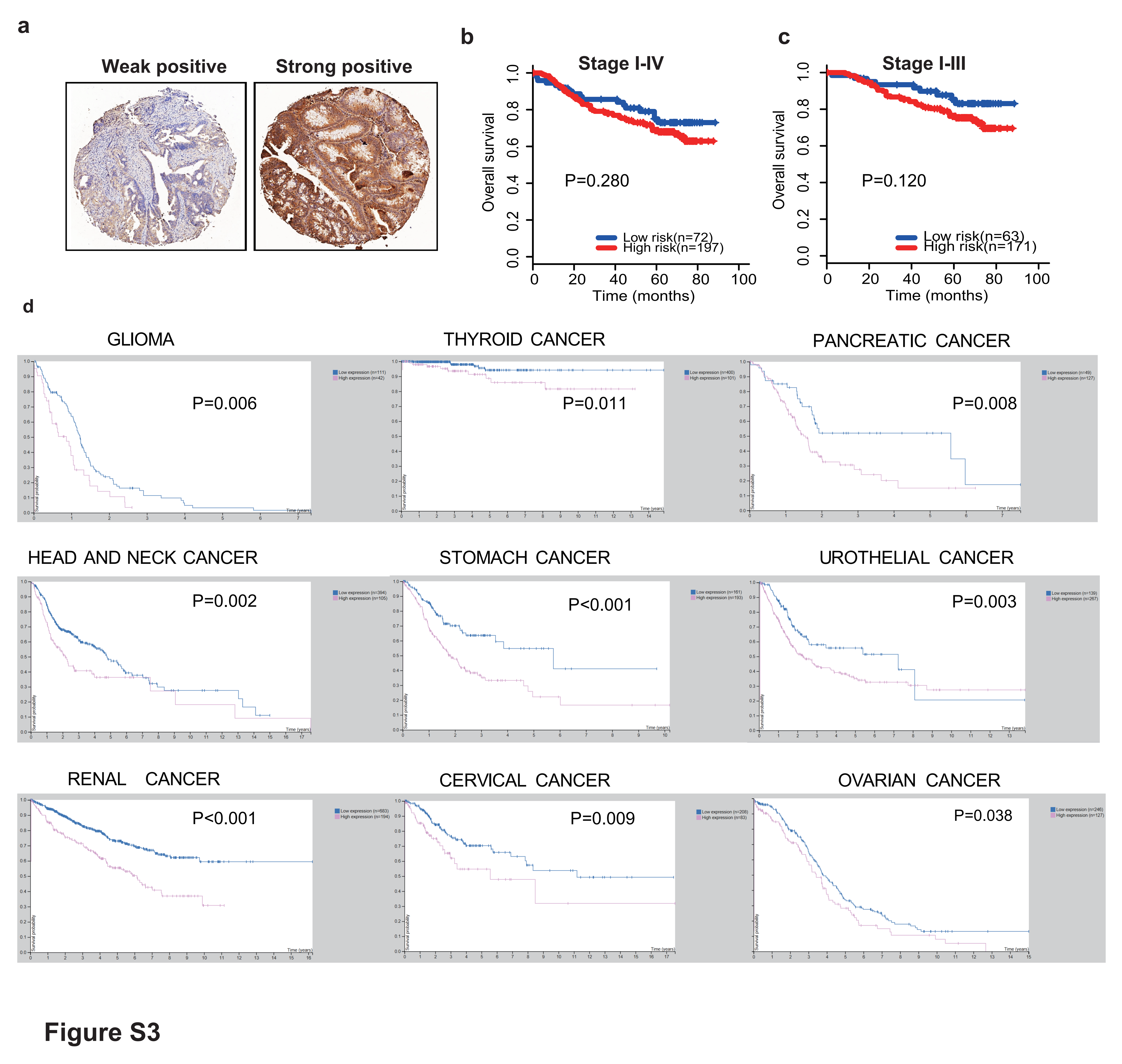
**Figure. S3.**

**Figure S3. Related to Figure 1.**

(a) Representative immunohistochemical staining for GLUT1 protein expression in CRC patients with different intensity.

(b) Kaplan-Meier analysis of GLUT1 expression and overall survival in the FUSCC cohort.

(c) GLUT3 is a general prognostic marker of solid tumors including stomach, pancreatic, renal, head and neck, ovarian, thyroid, urothelial, cervical and glioma cancers.


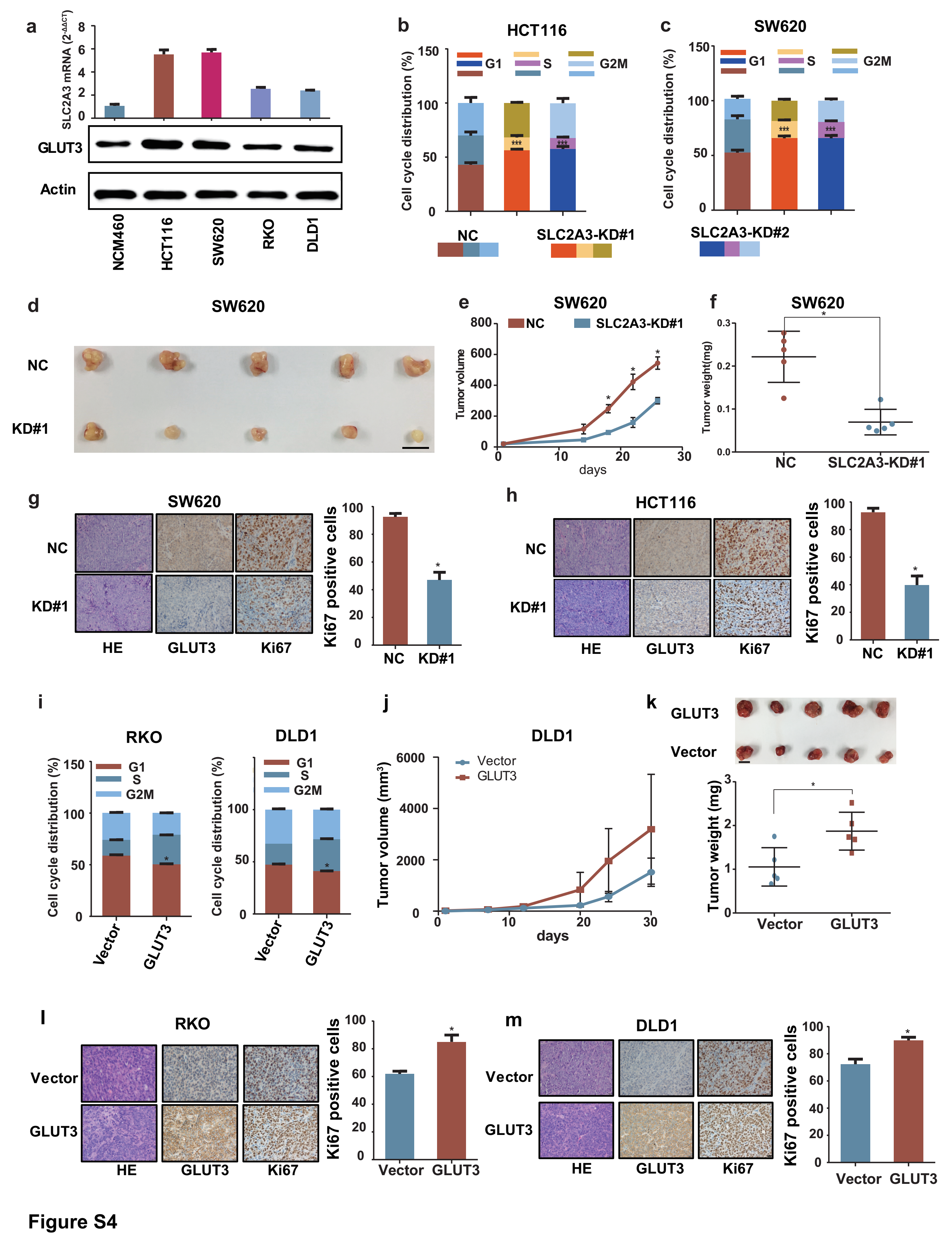
**Figure. S4.**

**Figure S4. Related to Figure 2 and Figure 3. GLUT3-mediated glucose utilization is essential for CRC growth *in vitro* and *in vivo*.**

(a) GLUT3 expression in five CRC cell lines determined using western bot and RT-PCR analysis.

(b-c) Cell cycle distribution of HCT116 and SW620 cells with or without SLC2A3 silence.

(d-f) Xenograft tumor images (D), subcutaneous tumor growth (E) and tumor weight (F) of SW620 cells with or without SLC2A3 silence in nude mice.

(g-h) Immunohistochemistry staining of Ki67 of HCT116 and SW620 xenografts without or without SLC2A3 silence.

(i) Cell cycle distribution of RKO and DLD1 cells with or without ectopic GLUT3 expression.

(j-k) Xenograft tumor images, subcutaneous tumor growth and tumor weight of DLD1 cells with or without enhanced GLUT3 expression in nude mice.

(l-m) Immunohistochemistry staining of Ki67 of RKO and DLD1 xenografts with or without ectopic GLUT3 expression.


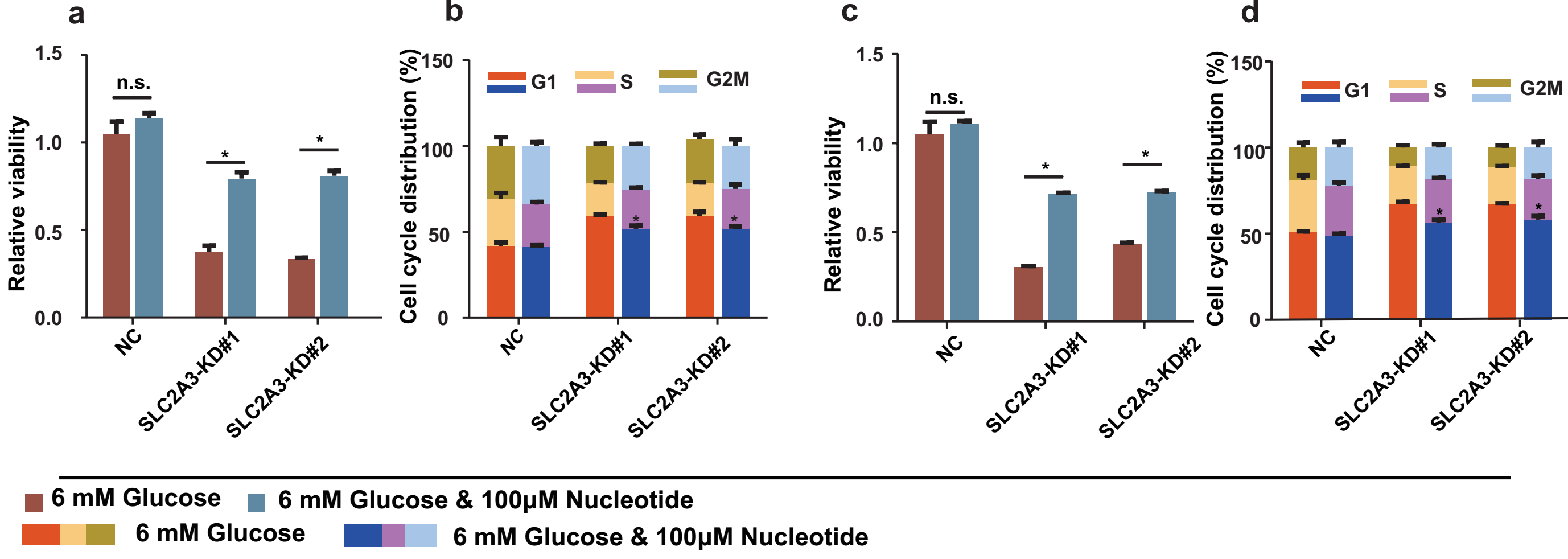
**Figure. S5.**

**Figure S5. Related to Figure 5. Cell proliferation and cell cycle analysis of nucleotides rescue in CRC cells with or without SLC2A3 silence.**

(a-b) Cell proliferation ability (A) and cell cycle distribution (B) of nucleotide rescue in HCT116 cells with or without attenuated GLUT3 expression.

(c-d) Cell proliferation ability (C) and cell cycle distribution (D) of nucleotide rescue in SW620 cells with or without GLUT3 knockdown.


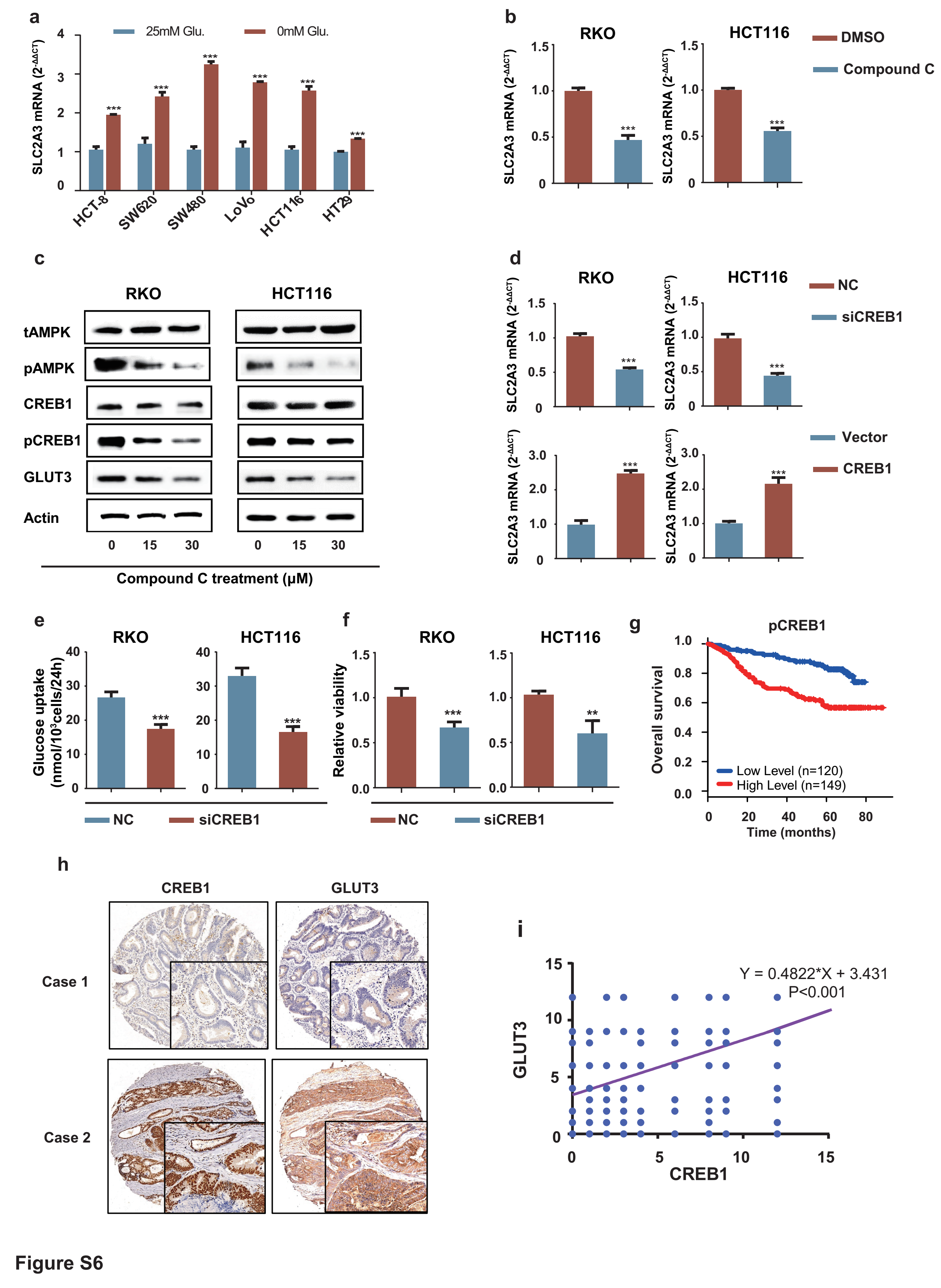
**Figure. S6.**

**Figure S6. Related to Figure 6. Activation of AMPK up-regulate GLUT3 expression and glucose uptake in CRC cells via phosphorylating CREB1.**

(a) mRNA expression of SLC2A3 in seven common used CRC cells cultured in 1 mM glucose-containing medium for 24 hours.

(b) mRNA expression of SLC2A3 in RKO and DLD1 cells treated with or without AMPK inhibitor Compound C.

(c) Western bot of CREB1, phosphorylated CREB1 and GLUT3 in RKO and DLD1 cells treated with or without AMPK inhibitor Compound C.

(d) mRNA expression of SLC2A3 in RKO and HCT116 cells treated with or without CREB1 siRNAs.

(e) The influence of ectopic attenuated CREB1 expression on glucose uptake of RKO and HCT116 cells.

(f) The influence of ectopic attenuated CREB1 expression on cell proliferation of RKO and HCT116 cells were cultured in complete medium containing 1 mM glucose for 48 hours.

(g) Kaplan-Meier analysis between pCREB1 expression and overall survival in the overall FUSCC cohorts

(h) Correlation analysis between pCREB1 and GLUT3 protein expression.

**
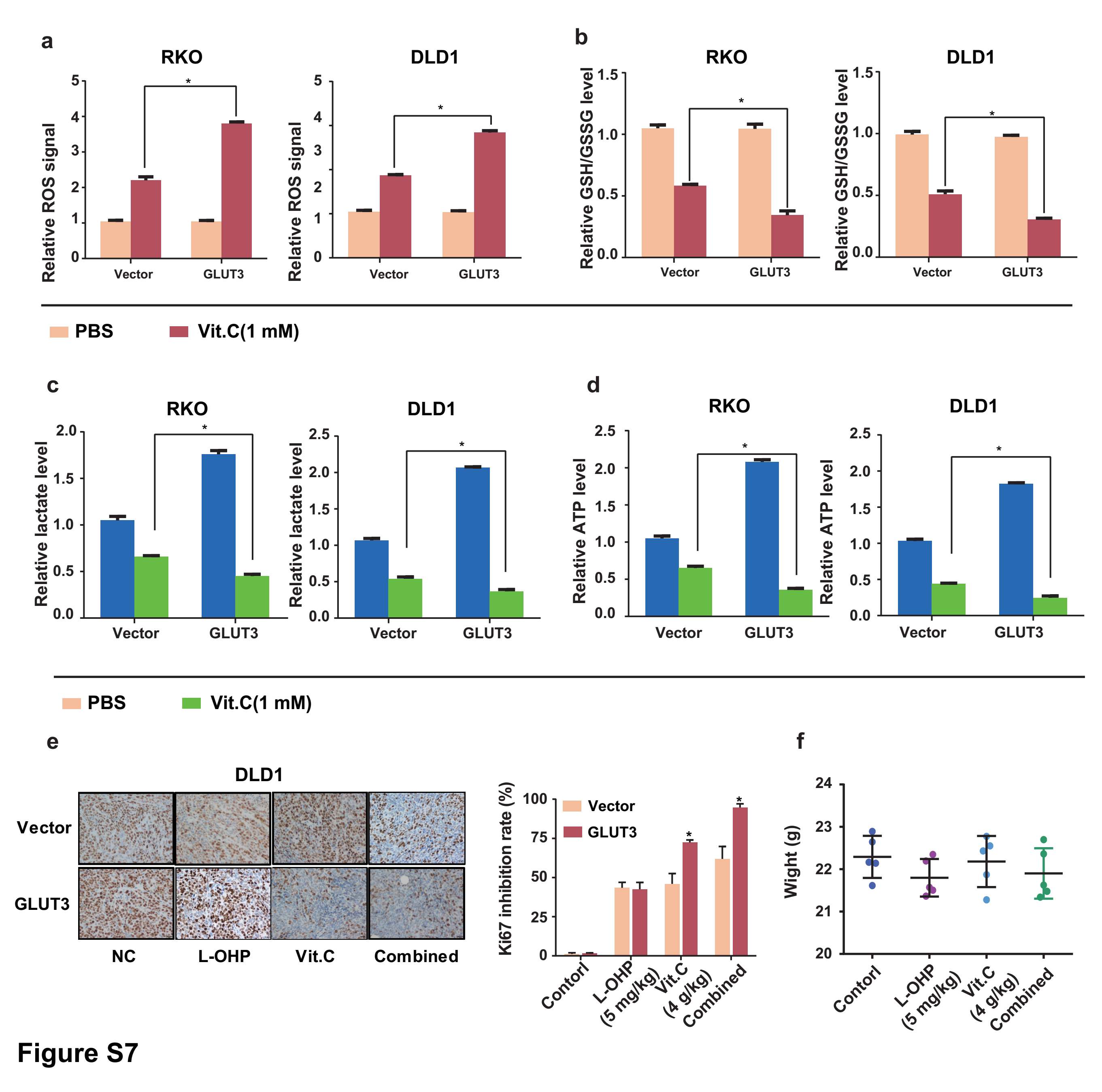
Figure. S7.**

**Figure S7. Related to Figure S7. Vitamin C is a safe and effective agent suitable for CRC patents with high expression of GLUT3.**

(a) ROS production of RKO and DLD1 cells with or without GLUT3 overexpression treated with Vitamin C.

(b) GSH/GSSG level of RKO and DLD1 cells with or without GLUT3 overexpression treated with Vitamin C.

(c-d) Lactate and ATP production of RKO and DLD1 cells with or without GLUT3 overexpression treated with Vitamin C.

(e) Immunohistochemistry staining of Ki67 of DLD1 xenografts with or without enhanced GLUT3 expression from nude mice treated with normal saline, single L-OHP, single Vitamin C and the combined two agents.

(f) Comparison of weight of mice treated with normal saline, single L-OHP, single Vitamin C and the combined two agents.

**Table S1.** Related to Figure 1. Relationship between GLUT3 expression and clinicopathological features.

|  | GLUT3 expression | | | |  |
| --- | --- | --- | --- | --- | --- |
|  | Low level | | High level | | P |
|  | N | % | N | % |  |
| Age |  |  |  |  | 0.468 |
| ≤60 | 68 | 64.80% | 99 | 60.40% |  |
| ＞60 | 37 | 35.20% | 65 | 39.60% |  |
| Sex |  |  |  |  | 0.354 |
| Female | 45 | 42.90% | 61 | 37.20% |  |
| Male | 60 | 57.10% | 103 | 62.80% |  |
| Stage |  |  |  |  | <0.001 |
| I | 16 | 15.20% | 11 | 6.70% |  |
| II | 49 | 46.70% | 38 | 23.20% |  |
| III | 36 | 34.30% | 84 | 51.20% |  |
| IV | 4 | 3.80% | 31 | 18.90% |  |
| Differentiation |  |  |  |  | <0.001 |
| Well / Morderate | 88 | 89.80% | 112 | 71.80% |  |
| Poor | 10 | 10.20% | 44 | 28.20% |  |
| Histological Type |  |  |  |  | 0.794 |
| Adenocarcinoma | 100 | 95.20% | 155 | 94.50% |  |
| Mucinous | 5 | 4.80% | 9 | 5.50% |  |
| Location |  |  |  |  | 0.01 |
| Left side | 71 | 67.62% | 85 | 51.83% |  |
| Right side | 34 | 32.38% | 79 | 48.17% |  |

**Table S2.** Related to Figure 1. Long-term survival of solid tumor patients with different expression level of *SLC2A1* and *SLC2A3* in TCGA database.

| Tumor type | SLC2A1 | | P^#^ | SLC2A3 | | P^#^ |
| --- | --- | --- | --- | --- | --- | --- |
|  | low level | high level |  | low level | high level |  |
| Glioma^*^ | 12% | 3% | 0.005 | 11% | 0% | 0.006 |
| Thyriod^†^ | 94% | 92% | 0.12 | 94% | 89% | 0.011 |
| Lung^†^ | 47% | 43% | <0.001 | 46% | 44% | 0.083 |
| Colorectal^†^ | 69% | 58% | 0.076 | 67% | 45% | <0.001 |
| Head & neck^†^ | 57% | 43% | 0.032 | 48% | 36% | 0.002 |
| Stomach^†^ | 44% | 30% | 0.11 | 55% | 22% | <0.001 |
| Liver^†^ | 55% | 34% | <0.001 | 50% | 44% | 0.11 |
| Pancreatic^†^ | 62% | 15% | <0.001 | 52% | 15% | 0.008 |
| Renal^†^ | 86% | 63% | <0.001 | 73% | 55% | <0.001 |
| Urothelial^†^ | 46% | 24% | <0.001 | 56% | 35% | 0.002 |
| Prostate^†^ | 98% | 97% | 0.19 | 97% | 100% | 0.054 |
| Testis^†^ | 99% | 94% | 0.095 | 99% | 95% | 0.17 |
| Breast^†^ | 82% | 80% | 0.036 | 79% | 83% | 0.092 |
| Cervical^†^ | 71% | 51% | 0.005 | 70% | 55% | 0.009 |
| Endometrial^†^ | 81% | 72% | 0.013 | 77% | 74% | 0.26 |
| Ovarian^†^ | 19% | 36% | 0.048 | 34% | 28% | 0.038 |
| Melanoma^*^ | 56% | 32% | 0.11 | 44% | 31% | 0.23 |

^†^5-year survival; ^*^3-year survival; ^#^Log rank test

**Table S3.** Related to Figure 6. Promoter sequence of *SLC2A3* in the transcription
activity assays.

| *SLC2A5* promoter | Species | Sequence (5'→3') |
| --- | --- | --- |
| Entire | Human | ACCCACCCTGCCACCTCCAGTACTGAAAGGGTTGCAAGCTACAAATGAATGGGCATTAGGAATTTGATAGTTGATATTCCTCCTCCAAAAAAGCCTAGATACTATCTCTCACTTACCCAGCTGTTTAAACACAAACCAAGTTGTTTTGCTTTGTTTACTCTCAACCTGGAACCACTCTTCTCTCTCCCCGATTATCCCTCCCTCAGTTCTCATTCCTATTTTCTTCTCCTGCTTAGCTTGTTCAGTTTTCTTGTATAGCTTAGTAATCAGATACTTATGTAACACTTTTCTTTCAAGAAACTCACAGTCCATAACTAATAGGATATTAATAATCTTTTCCAGTTATGTGAGAATTACTTGCCCTAGTCCCTTTTTATACTTTATAAAAAGGGAAGAAGGGAAACACTATAAATGACTTGTGGGTCAGAACTCTTTTGTCCTATTATTCTTTTTTTTTTTTTTTTTTGAGACGGATTCTTGCTCTGTCGCCCAGGCTGGAGTGCAGTGGCGCTGTCTTGGCTCACTGCAAGCTCCGCCTGCCGCGTTCACGCCATTCTCCTGTCTCAGCCTCCCGAGTAGCTGGGACTGCAGGCGCCCGCCACCATGCCCGGCTAATTTTTTTTTTTTTTTTTTTTTTTTTTGTATTTTTAGTAGAGAATGGTTTCACCGTGTTAGCCAGGATGGTCTTGATCTCCTGACCTCGTGATCCGCCCACCTCGGCCTCCCAAAGTGCTGGGATTACAGGCGTGAGCCACCGCGCCCGGCCTTTTTTTCTATTATTCACTGAAGAATCACCAGCTTCTTGGAGAGTACCTGATACATAGCGTGTATTCGGCAAATACCTGCTTAATTGAATTCGGTATTTATTGCATTTCTAGATGCTATAAGCTTGAGACTAGCAGAAAGTGATGCAGAATTCAGAATGGGTGATATGGCAAATGCCTTATTAAATATAGGGCTCGGCGCGGTGGCTCACACTTGTAATCCCAGCACTTTGGGAGGCCAAGGCGGGCAGATCACAAGGTCAGGAGTTTGAGACCAGCCTGGCCAATATGGTGAAACCCCGTCTTTACTAAAAATACAAAAATTAGCGGGGCGTGGTGGCGGGCGCCTATAGTCCCAGCTACTCGGGAGGCTGAGGCAGGAGAATTGCTTGAACCCGGGAGGCGGAGGTTGCAGTGAGCCGAGATCGCACCACTGCACTCCAGCCTGGGCGACAGAGAGAGACTCCATATCAAAAAAAAAAAAAAAAAAAAAAAAAGGATATGGATTGTATTCCATAAACAGTTGAACCTCTTATTCCTACTGCCCTGATAGTTGGTCTGGCTCCTAGAAATTCAGATTTTCTTCCCTCCAGGTGAGATTATGCGAAGAAAGGAACACTGGCAAAATTATTTCCCTGATTTTCTTCTCTCAGGCTGTCTTTCCCTCCCCTTTAAGATTGTGTACTCCGTCTCAAAAAAAAAAGAAAAAAAAAAGATTGTAGACCCAGCCTATAAATTCATAGGATCAAGAATTCTTACTAACACTCTTTCTAACAACCTTAAATCTCTGATAGTAGCACCCACTTGACTTCGTTTTGTATGGATAGATTTATTCTAATCTCTCTGTAGATTGTTTTAATCCCAAGCAGATGCGAGAGCATAAAATAGTGTATTTGCCCTCCCTCCAGGCTTTTCTGGTAGTATTTTGTCTGTGAAACTAAGAGGCTCTAGCTGCTATTAGAAGAGGGAAGGAGTAAGGATGAGCTTTTGAAAAAAAAAAAAAAAAAAAAACCCAGGGTGGAGAGAGTGGAAGGATGTGGTTTTAAGAGAGGGG**GGAGGGAGGGCGTTATTGTCT**GTGGGGCGGGGGCGGGGGTAGTTCTGATAACACAGAATTCCGAGAGATCACAAGATTGCTTCAGGGGGGTGGGGTGGGGTGGGGTGGGGCTGGGGGCTTGTCGCCCTTTCAGGCTCCACCCTTTGCGGAGATTATAAATAGTCATGATCCCAGCGAGACCC |

**Table S4.** Supplemental resource table

| REAGENT or RESOURCE | SOURCE | IDENTIFIER |
| --- | --- | --- |
| Antibodies |  |  |
| GLUT3 | Santa Cruz | sc-74399 |
| GLUT1 | Abcam | ab40084 |
| phospho AMPK Thr172 | Cell Signaling Technology | 2535 |
| AMPK | Cell Signaling Technology | 2532 |
| phospho CREB1 Ser133 | Cell Signaling Technology | 9198 |
| CREB1 | Cell Signaling Technology | 9104 |
| Ki-67 | Cell Signaling Technology | 9449 |
| β-actin | Cell Signaling Technology | 4970 |
| Chemicals, Peptides, and Recombinant Proteins |  |  |
| [U-^13^C6] -D-glucose | Cambridge Isotope Laboratories | CLM-1396-1 |
| Oxaliplatin | Selleck | S1224 |
| Lipofectamine 3000 | Thermo Fisher Scientific | L3000008 |
| D-Glucose | Sigma | G7012-1KG |
| L-ascorbic acid | Sigma | A4544 |
| ES cell qualified nucleoside | Merck | ES-008-D |
| AICAR | MCE | HY-13417 |
| Compound C | MCE | HY-13418A |
| Dual-Luciferase® Reporter Assay System | Promega | E1910 |
| Oligonucleotides |  |  |
| CHIP primers for CREB1:  FWD: CCCAGGGTGGAGAGAGTGGAAG REV: TTATAATCTCCGCAAAGGGTGGAG | This study | N/A |
| q-PCR primers for huaman Actin: F:CACTCTTCCAGCCTTCCTTC R:GTACAGGTCTTTGCGGATGT | This study | N/A |
| q-PCR primers for huaman SLC2A3: F:GCTGGGCATCGTTGTTGGA R:GCACTTTGTAGGATAGCAGGAAG | This study | N/A |
| q-PCR primers for huaman SLC2A1: F:TCTGGCATCAACGCTGTCTTC R:CGATACCGGAGCCAATGGT | This study | N/A |
| q-PCR primers for huaman SLC2A2: F:GCTGCTCAACTAATCACCATGC R:TGGTCCCAATTTTGAAAACCCC | This study | N/A |
| q-PCR primers for huaman SLC2A4: F:ATCCTTGGACGATTCCTCATTGG R:CAGGTGAGTGGGAGCAATCT | This study | N/A |
| q-PCR primers for huaman CREB1: F:CCACTGTAACGGTGCCAACT R:GCTGCATTGGTCATGGTTAATGT | This study | N/A |
| Critical Commercial Assays |  |  |
| FITC Annexin V Apoptosis Detection Kit I (RUO) | BD | 556547 |
| Cell Counting Kit-8(CCK-8) | Dojindo | CK04 |
| Cell Cycle Staining Kit | MULTI SCIENCES | CCS012 |
| Lactate Assay kit | Abcam | ab65330 |
| Glucose (HK) Assay Kit | Sigma | GAHK20-1KT |
| SYBR® Premix Ex Taq™ (Tli RNase H Plus) | Takara | RR42WR |
| Chromatin Immunoprecipitation Kit | Cell Signaling Technology | 9003 |
| GSH-Glo™ kit | Promega | V6911 |
| Luminescent ATP Detection Assay | Abcam | ab113849 |
| Experimental Models: Cell Lines |  |  |
| NCM460 | NCI | N/A |
| RKO | NCI | N/A |
| SW620 | NCI | N/A |
| HCT116 | NCI | N/A |
| DLD1 | NCI | N/A |
| HCT-8 | NCI | N/A |
| Experimental Models: Organisms/Strains |  |  |
| Mouse: BALB/c-nude | Shanghai SLAC Laboratory Animal Co.,Ltd | N/A |
| Recombinant DNA |  |  |
| psPAX2 packaging vector | Addgene | 12260 |
| pMD2.G packaging vector | Addgene | 12259 |
| pGL3-Basic vector | Promega | N/A |
| pGL3-Basic-human entire *SLC2A3* promoter | This study | N/A |
| pCDH-CMV-MCS-EF1-Puro vector | Promega | N/A |
| pCDH-CMV-MCS-EF1-Puro-human *SLC2A3* | This study | N/A |
| pCDH-CMV-MCS-EF1-Puro-human *CREB1* | This study | N/A |
| Renilla luciferase reporter vector pRL-SV40 | Promega | N/A |
| Software and Algorithms |  |  |
| R language | Open source | https://www.r-project.org/ |
| ImageJ software | Open source | https://imagej.nih.gov/ij/ |
| CRISPR Guide RNA database |  |  |
| Others |  |  |
| Dialyzed, fetal bovine serum | Thermo Fisher Scientific | 26400044 |
| DMEM, no Glucose | Thermo Fisher Scientific | 11966025 |

**Data S1.** Changes in metabolites influenced by GLUT3 expression.
